# Identification of a small molecule as inducer of ferroptosis and apoptosis through ubiquitination of GPX4 in triple negative breast cancer cells

**DOI:** 10.1186/s13045-020-01016-8

**Published:** 2021-01-20

**Authors:** Yahui Ding, Xiaoping Chen, Can Liu, Weizhi Ge, Qin Wang, Xin Hao, Mengmeng Wang, Yue Chen, Quan Zhang

**Affiliations:** 1 State Key Laboratory of Medicinal Chemical Biology, College of Pharmacy and Tianjin Key Laboratory of Molecular Drug Research, Nankai University, Haihe Education Park, 38 Tongyan Road, Tianjin, 300353 People’s Republic of China; 2 Accendatech Company, Ltd., Tianjin, 300384 People’s Republic of China

**Keywords:** GPX4, Ubiquitination, EGR1, Ferroptosis, Triple negative breast cancer

## Abstract

**Background:**

TNBC is the most aggressive breast cancer with higher recurrence and mortality rate than other types of breast cancer. There is an urgent need for identification of therapeutic agents with unique mode of action for overcoming current challenges in TNBC treatment.

**Methods:**

Different inhibitors were used to study the cell death manner of **DMOCPTL**. RNA silencing was used to evaluate the functions of GPX4 in ferroptosis and apoptosis of TNBC cells and functions of EGR1 in apoptosis. Immunohistochemical assay of tissue microarray were used for investigating correlation of GPX4 and EGR1 with TNBC. Computer-aided docking and small molecule probe were used for study the binding of **DMOCPTL** with GPX4.

**Results:**

**DMOCPTL**, a derivative of natural product parthenolide, exhibited about 15-fold improvement comparing to that of the parent compound PTL for TNBC cells. The cell death manner assay showed that the anti-TNBC effect of **DMOCPTL** mainly by inducing ferroptosis and apoptosis through ubiquitination of GPX4. The probe of **DMOCPTL** assay indicated that **DMOCPTL** induced GPX4 ubiquitination by directly binding to GPX4 protein. To the best of our knowledge, this is the first report of inducing ferroptosis through ubiquitination of GPX4. Moreover, the mechanism of GPX4 regulation of apoptosis is still obscure. Here, we firstly reveal that GPX4 regulated mitochondria-mediated apoptosis through regulation of EGR1 in TNBC cells. Compound **13**, the prodrug of **DMOCPTL**, effectively inhibited the growth of breast tumor and prolonged the lifespan of mice in vivo, and no obvious toxicity was observed.

**Conclusions:**

These findings firstly revealed novel manner to induce ferroptosis through ubiquitination of GPX4 and provided mechanism for GPX4 inducing mitochondria-mediated apoptosis through up-regulation of EGR1 in TNBC cells. Moreover, compound **13** deserves further studies as a lead compound with novel mode of action for ultimate discovery of effective anti-TNBC drug.

## Introduction

Ferroptosis is a new form of programmed cell death characterized by the accumulation of iron-dependent lipid peroxides, and is morphologically, biochemically, and genetically distinct from apoptosis, necrosis, and autophagy [1]. In recent years, more and more research revealed that ferroptosis was implicated in various human diseases, including Alzheimer’s, Parkinson’s and Huntington’s diseases, stroke, periventricular leukomalacia (PVL), intracerebral hemorrhage, kidney degeneration [2, 3]. Moreover, emerging evidence indicated that ferroptosis may also have a tumor-suppressor function for cancer therapy. It was reported that many cancer cells, such as liver cancer, diffuse large B cell lymphomas (DLBCL), renal cell carcinoma, gastric cancer, rhabdomyosarcoma, ovarian cancer cells, are susceptible to ferroptosis [4, 5]. Small molecule ferroptosis inducers had a strong inhibition of tumor growth and enhanced the sensitivity of chemotherapeutic drugs [6]. Combination of chemotherapeutic drugs such as tmozolomide, cisplatin, doxorubicin with ferroptosis inducer erastin resulted in a remarkable synergistic effect on tumor treatment [3]. Therefore, induction of ferroptosis has become a novel potential therapeutic strategy for cancer therapy [7–12].

Breast cancer is the most commonly being diagnosed cancer and the leading cause of cancer death for women. Worldwide, there are about 2.1 million newly diagnosed female breast cancer cases, and the incidence and mortality of female breast cancer is 24.2% and 15%, respectively, in 2018 [13]. Breast cancer accounts for 30% all new cancer diagnoses in women in USA [14]. Although 5-year relative survival rate for breast cancer is 90% in USA [14], the prognosis is still far from satisfactory, especially for triple-negative breast cancer (TNBC). TNBC is the most aggressive breast cancer with higher recurrence and mortality rate than other types of breast cancer [15]. TNBC represents 15–20% of breast carcinomas and is characterized by lack of expression of estrogen receptors (ER), progesterone receptors (PR) and human epidermal growth factor receptor 2 (HER-2) [16, 17]. TNBC patients cannot benefit from targeted therapy, such as endocrine or anti-HER2 therapy and chemotherapy is still the only validated therapy option for treatment of TNBC in clinical practice [18]. Therefore, there is an urgent need for identification of therapeutic agents with unique mode of action for overcoming current challenges in TNBC treatment.

It was reported that ferroptosis inducer erastin could selectively target MDA-MB-231 cells and efficiently induce ferroptosis in TNBC cells [19]. Sulfasalazine could induce ferroptosis in breast cancer cells with low ER expression [20]. Combination of siramesine and lapatinib induced ferroptosis in MDA-MB-231 and SKBR3 cells [21]. Kufe et al., identified that targeting MUC1-C/xCT signaling pathway induced ferroptosis of TNBC cells [22]. Lee and Tseng et al., reported that cystine starvation could trigger ferroptosis through CHAC1 degradation of glutathione via GCN2-eIF2α-ATF4 pathway in human TNBC cells [23]. Therefore, inducing ferroptosis is a new effective strategy for treatment of TNBC.

**Fig. 1:**
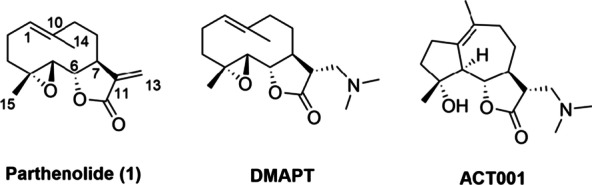
Structures of parthenolide (**1**), DMAPT, and ACT001

Sesquiterpene lactones (SLs) have attracted extensive attention because of their potent bioactivity, such as anti-cancer, anti-inflammatory and anti-malaria [24, 25]. Parthenolide (**1**, PTL, Fig. 1), a prominent germacrane-type SL, showed promising anti-cancer property. Nevertheless, PTL has some disadvantages, such as poor oral bioavailability, unstable under chemical and physiological conditions and poor water solubility [26, 27]. DMAPT, a derivative of PTL, effectively increased the water solubility and oral bioavailability, which has advanced into a phase I clinical trial for treatment of acute myeloid leukemia (AML) [25]. Recently, we developed the other PTL derivative, ACT001, to be in clinical trial in Australia and China for treatment of glioblastoma [28].

**Fig. 2:**
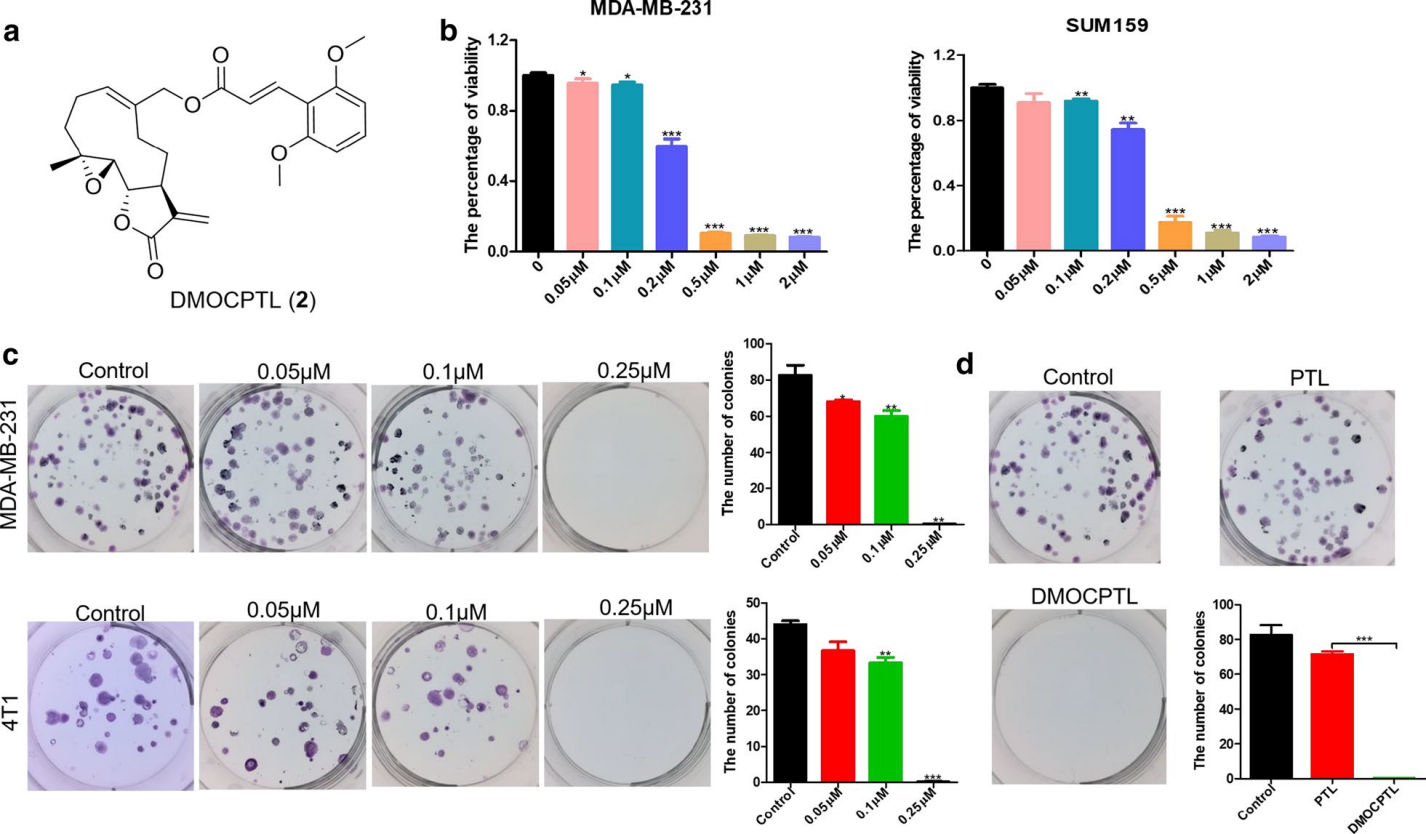
The antiproliferative activity of DMOCPTL in TNBC cells. **a** The chemical structure of **DMOCPTL**. **b** The effect of **DMOCPTL** on the viability of MDA-MB-231 and SUM159 cells after being treated for 72 h. **c** The representative pictures and colony number of MDA-MB-231 and 4T1 cells after treatment of **DMOCPTL** at concentrations of 0.05 μM, 0.1 μM and 0.25 μM, respectively. **d** The effect of **DMOCPTL** and PTL at a concentration of 0.5 μM on colony formation in MDA-MB-231 cells. Analysis results represented mean ± SD, *P < 0.05, **P < 0.01, ***P < 0.001

As previous studies suggested that GPX4 inhibition induced ferroptosis [29], we rationalized that an alternative way to lead to cancer cell death by inducing ferroptosis would be through ubiquitination of GPX4 to reduce abundance of GPX4. In this study, we revealed a PTL derivative (**2**, DMOCPTL, Fig. 2) as an inducer of ferroptosis and apoptosis through ubiquitination of GPX4 and a potential therapeutic agent for TNBC. To the best our knowledge, herein, this is the first report of inducing ferroptosis through ubiquitination of GPX4.

## Materials and methods

### Chemistry

**Synthesis of compound 4.** To a solution of **3** (500 mg, 2.74 mmol) and TBSCl (455 mg, 3.0 mmol) in CH_2_Cl_2_ was added imidazole (204 mg, 3.0 mmol) at 0 °C. The mixture was stirred overnight, quenched with NH_4_Cl, and extracted with CH_2_Cl_2_. The CH_2_Cl_2_ layer was dried over anhydrous Na_2_SO_4_ and concentrated under reduced pressure to provide **4** (731 mg, 2.47 mmol, yield 90%).

**Synthesis of compound 5**. To a solution of trimethyl phosphonoacetate (1.48 mL, 9.17 mmol) in anhydrous THF (20 mL) was added LiHMDS (1.0 M, 9.17 mL, 9.17 mmol) at − 20 °C. After 1.5 h, the mixture was cooled to − 78 °C. Compound **4** (1.36 g, 4.59 mmol) in THF was added to the mixture. The reaction mixture was quenched with saturated NaCl and extracted with ethylacetate. The organic layer was dried over anhydrous Na_2_SO_4_ and concentrated under reduced pressure to give a crude oil. The resulting oil was dissolved in THF, which was added TBAF in THF (5 mL, 1 N). After 12 h, the reaction was quenched with NH_4_Cl, extracted with ethylacetate. The organic layer was dried over anhydrous Na_2_SO_4_ and concentrated under reduced pressure to give an oil, which was purified with silica gel column chromatography to provide a white solid **5** (915 mg, yield 84%). ^1^H NMR (400 MHz, MeOD-*d*_4_) *δ* 8.19 (d, *J* = 16.2 Hz, 1H), 6.79 (d, *J* = 16.2 Hz, 1H), 6.20 (s, 2H), 3.95 (s, 6H), 3.86 (s, 3H). ^13^C NMR (100 MHz, MeOD-*d*_4_) *δ* 171.3, 162.4, 162.3, 137.4, 115.7, 104.9, 92.5, 55.9, 51.7. HRMS (ESI) cald for C_12_H_15_O_5_ [M + H]^+^ 239.0914, found 239.0910.

**Synthesis of compounds 6a-6e**. A mixture of **5** (100 mg, 0.42 mmol, 1 eq), K_2_CO_3_ (232.1 mg, 1.68 mmol, 4 eq) and different alkynyl bromide (1.47 mmol, 3.5 eq) in 4 mL anhydrous DMF was heated to 40 °C for 4 h. The reaction was quenched with NaCl and extracted with ethylacetate. The organic layer was dried over anhydrous Na_2_SO_4_ to give a crude oil, which was purified with silica gel column chromatography to provide white solid **6a-6e** in yields of 67%–88%.

**Synthesis of compound 8a**. A mixture of **6a** (110 mg, 0.4 mmol), LiOH•H_2_O (336 mg, 8 mmol, 20 eq) and THF–H_2_O (1:1) (4 mL) was stirred at 40 °C for 12 h. The pH of the mixture adjusted to 2–3 with 2 N HCl. The resulting mixture was extracted with ethylacetate. The organic layer was dried over anhydrous Na_2_SO_4_ and concentrated under reduced pressure to give a crude oil. The mixture of the crude oil, **7** (211 mg, 0.8 mmol, 2 eq), EDCI (155 mg, 0.8 mmol, 2 eq), DMAP (1.2 mg, 0.01 mmol), and Et_3_N (110.9 μL, 0.8 mmol, 2 eq) in 1 mL CH_2_Cl_2_ was stirred at room temperature for 12 h and quenched with sat. NaHCO_3_. The resulting mixture was extracted with CH_2_Cl_2_. The CH_2_Cl_2_ layer was dried over anhydrous Na_2_SO_4_ to give a crude oil, which was purified by silica gel column chromatography to provide a white solid **8a** (109 mg, 0.22 mmol, 54% for two steps). ^1^H NMR (400 MHz, CDCl_3_) *δ* 8.10 (d, *J* = 16.2 Hz, 1H), 6.71 (d, *J* = 16.2 Hz, 1H), 6.20 (d, *J* = 5.2 Hz, 3H), 5.74 (t, *J* = 8.1 Hz, 1H), 5.53 (d, *J* = 3.1 Hz, 1H), 4.73 (q, *J* = 4.9 Hz, 3H), 4.60 (d, *J* = 12.4 Hz, 1H), 3.95–3.75 (m, 7H), 3.20–3.04 (m, 1H), 2.94 (d, *J* = 9.4 Hz, 1H), 2.58 (t, *J* = 2.3 Hz, 1H), 2.54–2.13 (m, 6H), 1.65 (dd, *J* = 19.5, 6.7 Hz, 1H), 1.60–1.54 (m, 3H), 1.14 (t, *J* = 12.6 Hz, 1H). ^13^C NMR (100 MHz, CDCl_3_) *δ* 169.7, 168.8, 161.4, 161.0, 138.8, 136.4, 135.6, 131.0, 120.6, 116.9, 106.4, 100.1, 91.4, 81.2, 78.0, 76.3, 67.1, 63.5, 60.1, 56.0, 55.9, 42.9, 36.8, 26.3, 25.3, 24.0. HRMS (ESI) cald for C_29_H_32_NaO_8_ [M + Na]^+^ 531.1989, found 531.1992.

**8b** (white solid, 30%) was synthesized following the similar procedure for **8a**. ^1^H NMR (400 MHz, CDCl_3_) *δ* 8.11 (d, *J* = 16.2 Hz, 1H), 6.70 (d, *J* = 16.2 Hz, 1H), 6.20 (d, *J* = 3.2 Hz, 1H), 6.12 (s, 2H), 5.74 (t, *J* = 8.1 Hz, 1H), 5.53 (d, *J* = 2.8 Hz, 1H), 4.73 (d, *J* = 12.5 Hz, 1H), 4.60 (d, *J* = 12.3 Hz, 1H), 4.13 (t, *J* = 7.0 Hz, 2H), 3.93–3.77 (m, 7H), 3.13 (t, *J* = 9.6 Hz, 1H), 2.94 (d, *J* = 9.4 Hz, 1H), 2.77–2.63 (m, 2H), 2.55–2.13 (m, 6H), 2.07 (s, 1H), 1.70–1.63 (m, 1H), 1.25 (s, 3H), 1.14 (t, *J* = 12.5 Hz, 1H). ^13^C NMR (100 MHz, CDCl_3_) *δ* 169.7, 168.8, 162.0, 161.5, 138.9, 136.5, 135.7, 131.1, 131.0, 129.0, 120.5, 116.6, 91.1, 81.3, 70.3, 67.1, 66.2, 63.5, 60.1, 55.9, 43.0, 36.8, 26.4, 25.3, 24.1, 19.7, 18.2. HRMS (ESI) cald for C_30_H_34_NaO_8_ [M + Na]^+^ 545.2146, found 545.2148.

**8c** (white solid, 44%) was synthesized following the similar procedure for **8a**. ^1^H NMR (400 MHz, CDCl_3_) *δ* 8.09 (d, *J* = 16.2 Hz, 1H), 6.68 (d, *J* = 16.2 Hz, 1H), 6.18 (s, 1H), 6.10 (s, 2H), 5.72 (t, *J* = 8.0 Hz, 1H), 5.52 (s, 1H), 4.71 (d, *J* = 12.4 Hz, 1H), 4.59 (d, *J* = 12.4 Hz, 1H), 4.10 (t, *J* = 5.9 Hz, 2H), 3.85 (s, 7H), 3.10 (t, *J* = 9.4 Hz, 1H), 2.92 (d, *J* = 9.4 Hz, 1H), 2.53–2.10 (m, 8H), 2.01 (dd, *J* = 12.0, 5.4 Hz, 3H), 1.68–1.60 (m, 1H), 1.54 (s, 3H), 1.12 (t, *J* = 12.8 Hz, 1H). ^13^C NMR (100 MHz, CDCl_3_) *δ* 169.6, 168.8, 162.5, 161.5, 138.8, 136.5, 135.6, 130.8, 120.4, 116.3, 105.7, 91.0, 83.3, 81.2, 69.2, 67.0, 66.3, 63.4, 60.1, 55.8, 42.9, 36.8, 28.1, 26.3, 25.2, 24.0, 18.1, 15.2. HRMS (ESI) cald for C_31_H_36_NaO_8_ [M + Na]^+^ 559.2302, found 559.2305.

**8d** (white solid, 64%) was synthesized following the similar procedure for **8a**. ^1^H NMR (400 MHz, CDCl_3_) *δ* 8.10 (d, *J* = 16.2 Hz, 1H), 6.68 (d, *J* = 16.2 Hz, 1H), 6.19 (d, *J* = 3.4 Hz, 1H), 6.09 (s, 2H), 5.73 (t, *J* = 8.1 Hz, 1H), 5.52 (d, *J* = 3.0 Hz, 1H), 4.72 (d, *J* = 12.4 Hz, 1H), 4.59 (d, *J* = 12.4 Hz, 1H), 4.02 (t, *J* = 6.2 Hz, 2H), 3.86 (d, *J* = 13.4 Hz, 7H), 3.18–3.04 (m, 1H), 2.93 (d, *J* = 9.4 Hz, 1H), 2.55–2.12 (m, 8H), 1.97 (t, *J* = 2.3 Hz, 1H), 1.96–1.88 (m, 2H), 1.72 (dt, *J* = 14.7, 7.4 Hz, 2H), 1.64 (dd, *J* = 18.3, 6.8 Hz, 1H), 1.55 (s, 3H), 1.13 (t, *J* = 12.7 Hz, 1H). ^13^C NMR (100 MHz, CDCl_3_) *δ* 169.7, 168.8, 162.6, 161.5, 138.9, 136.6, 135.7, 130.9, 120.5, 116.3, 105.6, 91.0, 84.0, 81.2, 68.9, 67.6, 67.0, 63.5, 60.1, 55.9, 42.9, 36.8, 28.3, 26.3, 25.3, 25.1, 24.0, 18.3, 18.2. HRMS (ESI) cald C_32_H_38_NaO_8_ [M + Na]^+^ for 573.2459, found 573.2462.

**8e** (white solid, 42%) was synthesized following the similar procedure for **8a**. ^1^H NMR (400 MHz, CDCl_3_) *δ* 8.11 (d, *J* = 16.1 Hz, 1H), 6.69 (d, *J* = 16.2 Hz, 1H), 6.20 (s, 1H), 6.09 (s, 2H), 5.74 (t, *J* = 7.8 Hz, 1H), 5.53 (s, 1H), 4.72 (d, *J* = 12.4 Hz, 1H), 4.60 (d, *J* = 12.5 Hz, 1H), 4.00 (t, *J* = 6.1 Hz, 2H), 3.87 (d, *J* = 13.5 Hz, 6H), 3.12 (t, *J* = 9.3 Hz, 1H), 2.94 (d, *J* = 9.6 Hz, 1H), 2.50–2.12 (m, 8H), 1.96 (s, 1H), 1.89–1.75 (m, 2H), 1.64 (m, 6H), 1.55 (s, 3H), 1.14 (t, *J* = 12.4 Hz, 1H). ^13^C NMR (100 MHz, CDCl_3_) *δ* 169.7, 168.9, 162.7, 161.5, 138.9, 136.7, 135.7, 130.9, 120.5, 116.2, 105.6, 91.0, 84.4, 81.2, 68.6, 68.0, 67.0, 63.5, 60.1, 55.9, 42.9, 36.8, 28.8, 28.3, 26.3, 25.3, 25.3, 24.0, 18.5, 18.2. HRMS (ESI) cald for C_33_H_40_NaO_8_ [M + Na]^+^ 587.2615, found 587.2618.

### Synthesis of 10a

A mixture of azide (80 mg, 0.097 mmol) and **8a** (60 mg, 0.118 mmol), CuSO_4_ (0.014 mmol), sodium ascorbate (0.014 mmol), *tert*-butanol (1 mL) and water (0.5 mL) was stirred overnight at room temperature. Then, 3 mL water was added and the reaction mixture was extracted (3 × 10 mL) with EtOAc. The combined organic layers were washed with saturated brine, dried over anhydrous Na_2_SO_4_, and concentrated to give crude product, which was purified on a silica gel column to give compound **10a**.

^1^H NMR (400 MHz, MeOD) δ 8.15 (s, 1H), 8.06 (d, *J* = 16.2 Hz, 1H), 7.77 (s, 2H), 7.69 (s, 1H), 7.51 (s, 1H), 7.26 (d, *J* = 9.3 Hz, 2H), 7.05 (dd, *J* = 9.6, 2.4 Hz, 2H), 6.97–6.91 (m, 2H), 6.66 (d, *J* = 16.2 Hz, 1H), 6.33 (s, 2H), 5.70 (t, *J* = 8.3 Hz, 1H), 5.59 (d, *J* = 3.2 Hz, 1H), 5.24 (s, 2H), 4.73–4.61 (m, 2H), 4.62–4.57 (m, 2H), 3.85 (s, 6H), 3.67 (m, 10H), 3.62–3.56 (m, 4H), 3.54 (d, *J* = 10.5 Hz, 7H), 3.46 (t, *J* = 5.5 Hz, 4H), 3.34 (s, 7H), 2.92 (d, *J* = 9.5 Hz, 1H), 2.53 (s, 2H), 2.41 (d, *J* = 6.9 Hz, 3H), 2.37–2.25 (m, 2H), 2.23–2.08 (m, 3H), 1.55 (s, 4H), 1.29–1.28 (m, 15H).

### Synthesis of compound 12 in two methods

**Method A**: A solution of **7** (1.1 g, 4.16 mmol) in dimethylamine (20.8 mL, 41.6 mmol, 2 N in THF) was stirred for 1 h at 0 °C. The solvent was removed under reduced pressure. The residue was purified by silica gel column chromatography (CH_2_Cl_2_: MeOH = 50:1) to afford the desired product **11** (1.2 g, 93%) as a white solid. ^1^H NMR (400 MHz, CDCl_3_) δ 5.58 (t, *J* = 7.9 Hz, 1H), 4.10 (dd, *J* = 30.7, 13.1 Hz, 2H), 3.85 (t, *J* = 9.2 Hz, 1H), 3.38 (s, 1H), 2.81 (d, *J* = 9.4 Hz, 1H), 2.73 (dd, *J* = 12.9, 5.1 Hz, 1H), 2.61 (dd, *J* = 12.9, 5.4 Hz, 1H), 2.51–2.38 (m, 4H), 2.30–2.18 (m, 8H), 2.17–2.06 (m, 2H), 1.67–1.55 (m, 1H), 1.52 (s, 3H), 1.12–1.02 (m, 1H). ^13^C NMR (100 MHz, CDCl_3_) δ 177.0, 141.2, 127.5, 81.7, 66.3, 64.2, 60.0, 57.6, 45.8, 44.3, 42.1, 37.2, 27.7, 26.0, 23.9, 18.1. HRMS (ESI) calcd for C_17_H_28_N_2_O_4_ [M + H]^+^ 310.2013, found 310.2015.

To a solution of 2,6-dimethoxylcinnamic acid (712 mg, 3.42 mmol), compound **11** (705 mg, 2.28 mmol), EDCI (655.6 mg, 3.42 mmol) and DMAP (27.8 mg, 0.228 mmol) in 25 mL CH_2_Cl_2_ was added TEA (0.45 mL, 3.42 mmol) at 0 °C. The mixture was stirred for 8 h at room temperature. The reaction was quenched with saturated aqueous NaHCO_3_ and extracted with CH_2_Cl_2_ (3 × 75 mL). The combined organic layers were washed with saturated brine, dried over Na_2_SO_4_, and concentrated to give an oily crude product, which was purified on a silica gel column [DCM:MeOH = 50:1] to yield compound **12** (987.2 mg, 86%) as a white solid. ^1^H NMR (400 MHz, CDCl_3_) δ 8.16 (d, *J* = 16.3 Hz, 1H), 7.28 (d, *J* = 8.7 Hz, 1H), 6.89 (d, *J* = 16.3 Hz, 1H), 6.56 (d, *J* = 8.4 Hz, 2H), 5.67 (t, *J* = 7.8 Hz, 1H), 4.84 (d, *J* = 12.8 Hz, 1H), 4.66 (d, *J* = 12.8 Hz, 1H), 3.97–3.80 (m, 7H), 2.84 (d, *J* = 9.3 Hz, 1H), 2.73 (d, *J* = 4.3 Hz, 1H), 2.65 (d, *J* = 5.9 Hz, 1H), 2.58–2.26 (m, 6H), 2.23 (s, 6H), 2.15 (d, *J* = 12.5 Hz, 2H), 1.58 (d, *J* = 20.6 Hz, 4H), 1.10 (t, *J* = 12.8 Hz, 1H). ^13^C NMR (100 MHz, CDCl_3_) δ 177.2, 168.3, 160.2, 136.3, 136.1, 131.6, 128.5, 120.2, 112.2, 103.8, 81.3, 66.2, 64.0, 60.0, 58.5, 55.9, 45.9, 44.7, 43.3, 37.1, 27.2, 25.0, 23.9, 18.1. HRMS (ESI) calcd for C_28_H_38_NO_7_ [M + H]^+^ 500.2643, found 500.2644.

**Method B**. **Synthesis of compound 2.** To a mixture of **7** (53 mg, 0.2 mmol), EDCI (115 mg, 0.6 mmol), DMAP (1.2 mg, 0.01 mmol) and 2,6-dimethoxylcinnamic acid (0.3 mmol, 1.5 eq) in CH_2_Cl_2_ 2 mL was added triethylamine (83.4 μL, 0.6 mmol). After 12 h, saturated NaHCO_3_ was added. The resulting mixture was extracted with CH_2_Cl_2_. The CH_2_Cl_2_ layer was dried over anhydrous Na_2_SO_4_, and concentrated under reduced pressure to give crude product, which was purified by silica gel column chromatography to afford compound **2** (yield 83%). ^1^H NMR (400 MHz, CDCl_3_) *δ* 8.16 (d, *J* = 16.3 Hz, 1H, H-18), 7.30–7.22 (m, 1H, overlap with CHCl_3,_ H-4′), 6.83 (d, *J* = 16.3 Hz, 1H, H-17), 6.54 (d, *J* = 8.4 Hz, 2H, H-3′, H-5′), 6.19 (d, *J* = 3.5 Hz, 1H, H-13), 5.73 (t, *J* = 8.1 Hz, 1H, H-1), 5.53 (d, *J* = 3.2 Hz, 1H, H-13), 4.74 (d, *J* = 12.4 Hz, 1H, H-14), 4.60 (d, *J* = 12.4 Hz, 1H, H-14), 3.96–3.75 (m, 7H, H-6, H-19, H-18), 3.14–3.02 (m, 1H, H-7), 2.91 (d, *J* = 9.4 Hz, 1H, H-5), 2.49–2.11 (m, 6H, H-2, H-3, H-8, H-9), 1.71–1.60 (m, 1H, H-8), 1.54 (s, 3H, H-15), 1.12 (t, *J* = 12.6 Hz, 1H, H-3). ^13^C NMR (100 MHz, CDCl_3_) *δ* 169.6(C-12), 168.4(C-16), 160.2(C-2′, C-6′), 138.8(C-11), 136.5(C-18), 135.5(C-10), 131.7(C-4′), 130.9(C-1), 120.5(C-13), 119.5(C-17), 111.9(C-1′), 103.7(C-3′, 5′), 81.2(C-6), 67.1(C-14), 63.4(C-5), 60.1(C-4), 55.9(C-19, C-20), 42.8(C-7), 36.7(C-3), 26.2(C-8), 25.1(C-9), 24.0(C-2), 18.1(C-15). HRMS (ESI) cald for C_26_H_34_NO_7_ [M + NH_4_]^+^ 472.2330, found 472.2329.

A solution of compound **2** (930 mg, 2.05 mmol) and dimethylamine (10.5 mL, 21.0 mmol, 2 N in THF) at 0 °C was stirred 1 h at 0 °C. The solvent was removed under reduced pressure. The residue was purified by silica gel column chromatography (CH_2_Cl_2_: MeOH = 50:1) to afford the desired product **12** (985 mg, 96%) as a white solid.

**Synthesis of compound 13**. To a solution of compound **12** (1.15 g, 2.3 mmol) in methanol (23 mL), fumaric acid (267 mg, 2.3 mmol) was added. The mixture was stirred for 6.5 h and concentrated under vacuum to afford compound **13** as a white amorphous solid (1.3 g, yield 92%). ^1^H NMR (400 MHz, DMSO-*d*6) δ 12.95 (s, 2H), 8.00 (d, *J* = 16.3 Hz, 1H), 7.37 (t, *J* = 8.4 Hz, 1H), 6.78 (d, *J* = 16.3 Hz, 1H), 6.72 (d, *J* = 8.4 Hz, 2H), 6.61 (s, 2H), 5.59 (t, *J* = 7.6 Hz, 1H), 4.79 (d, *J* = 12.7 Hz, 1H), 4.57 (d, *J* = 12.6 Hz, 1H), 4.03 (t, *J* = 9.5 Hz, 1H), 3.86 (s, 6H), 2.77–2.54 (m, 4H), 2.39–2.23 (m, 4H), 2.18 (s, 6H), 2.10 (dd, *J* = 24.6, 10.8 Hz, 3H), 1.65 (t, *J* = 11.4 Hz, 1H), 1.48 (s, 3H), 0.94 (t, *J* = 12.3 Hz, 1H). ^13^C NMR (100 MHz, DMSO-*d*6) δ 177.1, 167.2, 166.2, 159.6, 135.8, 135.2, 134.2, 132.2, 128.1, 119.4, 110.7, 104.1, 80.4, 66.0, 63.2, 59.8, 57.9, 56.0, 45.2, 43.3, 42.6, 36.6, 25.6, 24.2, 23.1, 17.5. HRMS (ESI) calcd for C_28_H_38_NO_7_ [M + H]^+^ 500.2643, found 500.2645.

### Cell culture

Human triple negative breast cancer cell lines MDA-MB-231, SUM159, BT574, and mouse breast cancer cell 4T1 were purchased from ATCC and were cultured in 1640 medium supplement with 10% FBS under a 5% CO_2_ humidified atmosphere at 37 °C. Hs578T and MDA-MB-468 breast cancer cells were cultured in DMEM medium supplement with 10% FBS under a 5% CO_2_ humidified atmosphere at 37 °C.

### MTT assay

Human triple negative breast cancer cells were seeded into 96 well plate at the density of 4000 cells/200µL/well. After the adherent cell growth, **DMOCPTL** was added with a series concentration for 72 h. Then, 20 µL thiazolyl blue tetrazolium bromide (MTT, 5 mg/mL) was added and incubated for additional 4–6 h. The supernatant was discarded and the precipitate was dissolved with DMSO. Then, the absorbance at 570 nm was measured.

### Cell colony formation assay

Breast cancer cells MDA-MB-231 and 4T1 were seeded into 6 well plate with a concentration of 500 cells/1 mL/well. After 8–12 h, compounds at different concentration were added and incubated for 10 days. Then, the cells were fixed with 4% polyoxymethylene at room temperature for 15 min. After being washed with PBS, the cells were stained with crystal violet solution at room temperature for 15 min. The excess crystal violet was removed and washed with water for 3 times. The number of colonies was counted.

### Cell death manner assay

To investigate the mechanism of **DMOCPTL** in breast cancer proliferation inhibition, the manners of cells death were analyzed. The cell apoptosis inhibitor Z-VAD-FMK, cell autophagy inhibitor 3-methyladenine, cell necrosis inhibitor necrostatin-1, and ferroptosis inhibitors *N*-Acetyl-L-cysteine, deferoxamine and Ferrostatin-1 were purchased and stored at –20 °C. MDA-MB-231 cells were seeded with a density of at 5000 cells/200µL/well into 96 well plate. After 8–12 h, **DMOCPTL** was added at different concentrations. Following that 10 µM Z-VAD-FMK, 5 mM 3-methyladenine, 10 µM necrostatin-1, 3 mM N-acetyl-L-cysteine, 200 µM deferoxamine and 10 µM Ferrostatin-1 were added and co-incubated with **DMOCPTL**, respectively, for 72 h. Then, 20 µL thiazolyl blue tetrazolium bromide (MTT, 5 mg/mL) was added and incubated at 37 °C for additional 4 h. Then, supernatant was discarded and 200 μL DMSO was added to dissolve the precipitate. After 15 min, the absorbance was measured at 570 nm. The percentage of inhibition was calculated.

### Cell apoptosis assay

MDA-MB-231 and SUM159 cells were collected and washed with cold PBS. Then, the cells were suspended with 100 μL of binding buffer; 5 μL of Annexin V-APC and 5 μL PI were added and incubated for 15 min in dark at room temperature. Then, the cells were analyzed by flow cytometry within 1 h.

### Flow cytometry with reactive O_2_ species assay

MDA-MB-231 and SUM159 cells in logarithmic growth period were digested and divided into control group, **DMOCPTL**-treated group. The cells were washed with PBS for 3 times and **DMOCPTL** was added. Then, 10 µM DCF-DA or BODIPY 581/591 C11 were added and co-incubated with **DMOCPTL** in an incubator at 37 °C. The cells were collected by centrifugation and analyzed by flow cytometry within 1 h.

### Fe^2+^ intensity assay

MDA-MB-231 was collected and washed with cold PBS buffer. Then, cells were incubated with PGSK at 10 µM at 37 °C for 30 min in dark. Then, cells were collected, washed and analyzed by flow cytometry within 1 h.

### RNA interference

Breast cancer cell at about 40–50% confluence was transfected with siRNA using lipofectamine™ 2000 according to the manufacturer's instructions. After 48 h, the efficacy of siRNA was confirmed by western blot assay.

### Immunofluorescence assay

MDA-MB-231 cells were cultured on gelatin-coated glass coverslips for 24 h and treated with **DMOCPTL** for 48 h. Then, the cells were fixed in 4% paraformaldehyde for 20 min and permeabilized with Triton X-100 for 15 min. After blocked with horse serum for 30 min, the cells were incubated with GPX4 antibody over night at 4 °C. After the incubation, the cells were washed five times with PBST and incubated with the anti-rabbit IgG-FITC secondary antibody for 1 h at room temperature. Then, the cell nucleus was stained with DAPI and analyzed using fluorescence microscopy. As to flow cytometry assay, MDA-MB-231 cells with **DMOCPTL** treatment for 48 h were collected, fixed with 4% paraformaldehyde for 20 min, permeabilized with Triton X-100 for 15 min, incubated with GPX4 antibody overnight, then incubated with corresponding FITC-conjunct second antibody. Fluorescence intensity of GPX4 expression was analyzed by flow cytometry.

### Western blot assay

MDA-MB-231 and SUM159 cells were collected and lysed with RIPA buffer for 30 min on ice. Then, the protein (50 μg) from each sample were separated by 12% tris-acrylamide gel electrophoresis and transferred onto PVDF membrane. After blocking with 5% skim milk for 1 h at room temperature, Primary antibodies against GPX-4, EGR1, Bax, Bcl-2, Bcl-xl, cytochrome C, caspase 3, caspase 9 and PARP was used and incubated at 4 °C overnight on rotary shaker. After washing with PBST for 5 times, the membrane was probed with goat anti-rabbit IgG highly cross-adsorbed secondary antibody (1:10,000) for 2 h at room temperature. Then, the membrane was washed for 5 times and developed with ECL reagent.

### Probe pull-down assay

The pull-down experiments were performed using the probe of **DMOCPTL**. In vitro pull-down assay, MDA-MB-231 and SUM159 cells were collected and lysed, respectively, in RIPA buffer supplemented with protease inhibitors. Then, probe **10** was added and incubated for 2 h at room temperature with different concentrations. Then, excessive pre-cooled methanol was added and incubated at − 80 °C for 30 min to precipitate the protein. After centrifuged at 14,000×*g* for 15 min, the precipitated proteins were dissolved and incubated with streptavidin conjunct agarose A + G beads at 4 °C overnight. Then, the streptavidin beads were washed three times with PBS buffer, and the bead-bound proteins were collected and detected by western blot experiments.

As to in situ pull-down assay, MDA-MB-231 and SUM159 cells were seeded into 6 well plate. After 12 h, probe **8a** was added at different concentrations and incubated for additional 6 h. Then, the cells were collected and lysed, respectively, in RIPA buffer supplemented with protease inhibitors. After the centrifugation, the supernatant was collected and a mix of TBTA (0.1 mM), TCEP (1 mM), biotin-N_3_ (100 µM) and CuSO_4_ (1 mM) was added to conjunct biotin on probe **8a**. Then, excessive pre-cooled methanol was added and incubated at − 80 °C for 30 min to precipitate the protein. After centrifuged at 14,000×*g* for 15 min, the precipitated proteins were dissolved and incubated with streptavidin conjunct agarose A + G beads at 4 °C overnight. Then, the streptavidin beads were washed three times with PBS buffer, and the bead-bound proteins were collected and detected by western blot experiments or sliver staining.

### Co-localization assay

MDA-MB-231 cells were cultured on gelatin-coated glass coverslips for 24 h and treated with probe **10a** for 6 h at 2 µM. The cells were fixed in 4% paraformaldehyde for 20 min and permeabilized with Triton X-100 for 15 min. After being blocked with horse serum for 30 min, the cells were incubated with anti-GPX4 antibody (rabbit) and anti-Ubiquitin (mouse) over night at 4 °C. After the incubation, the cells were washed five times with PBST and incubated with the anti-rabbit IgG-FITC secondary antibody and anti-mouse IgG-AF647 secondary antibody for 2 h at room temperature. After being washed for 3 times, the cell nucleus was stained with DAPI and analyzed using fluorescence microscopy.

### Bioinformatics analysis

The relationship between GPX4 and gene functional states was analyzed by CancerSEA (http://biocc.hrbmu.edu.cn/CancerSEA/). The expression level of EGR1 in normal breast tissue and breast cancer tissues with different stages was analyzed in TCGA by UALCAN analysis (http://ualcan.path.uab.edu). The immunohistochemical staining intensity of EGR1 in normal breast tissue and breast cancer tissues with different stages were analyzed by Human Protein Atlas database (http://www.proteinatlas.org/).

### Docking simulations

Molecular Docking simulations were performed with the software of AutoDock. The crystal structure of the published GPX4 (PDB code: 5H5S) was retrieved from the RCSB Protein Data Bank. The solvent molecules within the protein structure were removed in the docking calculations, and the best ligand pose was chosen according to the dock score.

### In vitro ubiquitination assay

MDA-MB-231 cells in logarithmic growth period were plated into 6-well plate with the density at 1 × 10^5^ cells/mL/well. Then, **DMOCPTL** at the concentration of 0.05 µM, 0.1 µM, 0.2 µM, 0.5 µM and 1 µM were added and incubated for 24 h. Then, 10 µM MG-132 was added to inhibit the protein degradation for 4 h. After that the cells were collected by centrifugation and washed with PBS, subsequently suspended with RIPA buffer for 30 min on ice. After centrifugation, the GPX4 antibody was added with 1:100 dilution for 2 h on ice, then 10 µL agarose G was added and co-incubated at 4 °C overnight on rotary shaker. The samples were collected and washed with PBS for 3 times, then the SDS-PAGE protein electrophoresis was performed and the protein was transferred onto PVDF membrane. Following that the membrane was blocked in 5% skim milk for 1 h at room temperature, and subsequently was incubated with ubiquitin antibody (P4D1) at 4 °C overnight on rotary shaker.

### Immuno-coprecipitation assay

MDA-MB-231 and SUM159 cells were collected and lysed with RIPA buffer for 30 min on ice. The indicated protein in the cell lysates was immunoprecipitated using ubiquitin antibody. After incubated with ubiquitin antibody at 4 °C for 6 h, 10 µL agarose A + G was added and co-incubated at 4 °C overnight on rotary shaker. The samples were washed with PBS and visualized using an ECL detection system.

### Pharmacodynamics

Compound **13** was injected into three male SD rats by intravenous administration at a dose of 1 mg/kg. Then, the blood samples from jugular sinus of rats were collected at 2 min, 5 min, 15 min, 30 min, 1 h, 2 h, 3 h, 4 h, 6 h and 8 h after administration. After being centrifugated at 12000 rpm for 1 min, the supernatant was collected, respectively. Then, equal amount of acetonitrile was added into the sample, which was vortexed for 2 min, centrifugated at 12000 rpm for 5 min, and then the supernatant was collected and analyzed by LC/MS. The pharmacodynamic of **13** on SD rats was calculated by DAS 3.3.

### Acute toxicity assay in Bar b/c mice

To verify the toxicity of **13**, Bar b/c mice were administrated by intravenous administration at a dose of 50 mg/kg or oral administration with **13** at a dose of 500 mg/kg or vehicle control. During the experiment, their behavior was observed, and the body weight was recorded every day. The level of GPT, GOT and Cr in serum was detected by ELISA assay and the major organs of mice including liver, spleen, lung, kidney, heart, and brain were weighted, fixed with 4% paraformaldehyde and sectioned to 4 µm slides. After dewaxing and hydration, HE staining was performed. The histology and morphology were observed under microscope.

### In vivo anti-tumor activity assay

The experimental procedures of the animal experiments were permitted by the Animal Care and Use Committee at Nankai University. 4T1 triple negative breast cancer cells (1 × 10^5^) were injected on the Bar b/c mouse's breast fat pad. Then, compound **13** was administrated through intravenous injection (7.5 mg/kg) according to body weight. Body weights and tumor size were measured every other day. The tumor growth inhibition was calculated and the tumor weight was recorded when the mice were sacrificed. The tumor was collected and crushed with cell lysate buffer. Then, the expression of GPX4 and EGR1 were analyzed in vehicle and **13**-treated group by western blot assay. Furthermore, to analysis the effect of **13** on the overall survival compound **13** was administrated orally (50 mg/kg) according to body weight on tumor animal model. After administration for 6 times every other day the overall survival and body weight were recorded and analyzed.

### Immunohistochemical assay

The tumors isolated from the mice were fixed with 4% paraformaldehyde and sectioned to 4 µm slides. As to IHC assay, after dewaxing, hydration and antigen retrieval, the slides were incubated with primary antibodies at 4 °C overnight. After washed with PBST for 3 times, the slides were incubated with biotinylated secondary antibody for 1 h. Then, the slides were developed by DAB.

## Results

### DMOCPTL inhibited the antiproliferation of TNBC cells

Sesquiterpene lactone PTL showed moderate activity against breast cancer [30]. To improve the anticancer effect of parthenolide, we designed and synthesized a series of parthenolide derivatives. Ultimately, we identified that **DMOCPTL** exhibited the most potent anti-TNBC activity [31]. As shown in Fig. 2b, **DMOCPTL** significantly inhibited proliferation of MDA-MB-231 and SUM159 cells in a dose dependent manner. To further investigate the inhibitory effect of **DMOCPTL** on proliferation of TNBC cells, the colony formation of MDA-MB-231 and 4T1 cells assay was performed. After treatment of **DMOCPTL** for 10 days, the number of colonies was counted. The number of colonies was 82.7 ± 5.5, 68 ± 1, 60 ± 3 and 0 with treatment of **DMOCPTL** at different concentrations of 0, 0.05, 0.1 and 0.25 µM, respectively, in human breast cancer MDA-MB-231 cells. As to mouse breast cancer 4T1 cells, the number of colonies was 44 ± 1, 36.7 ± 2.5, 33.3 ± 1.5 and 0 with the treatment of **DMOCPTL** at 0, 0.05, 0.1 and 0.25 µM, respectively. Moreover, we compared the efficacy of **DMOCPTL** and PTL on inhibiting colony formation of MDA-MB-231 cells. As shown in Fig. 2d, the number of colonies was 82.7 ± 5.5, 71.5 ± 1.5 and 0 after treatment with vehicle, PTL or **DMOCPTL** at the same concentration of 0.5 µM. These results indicated that **DMOCPTL** could significantly inhibit colony formation of TNBC cells. Moreover, the inhibitory effect of **DMOCPTL** was evidently higher than that of PTL.

### DMOCPTL induced apoptosis and ferroptosis of TNBC cells

**Fig. 3:**
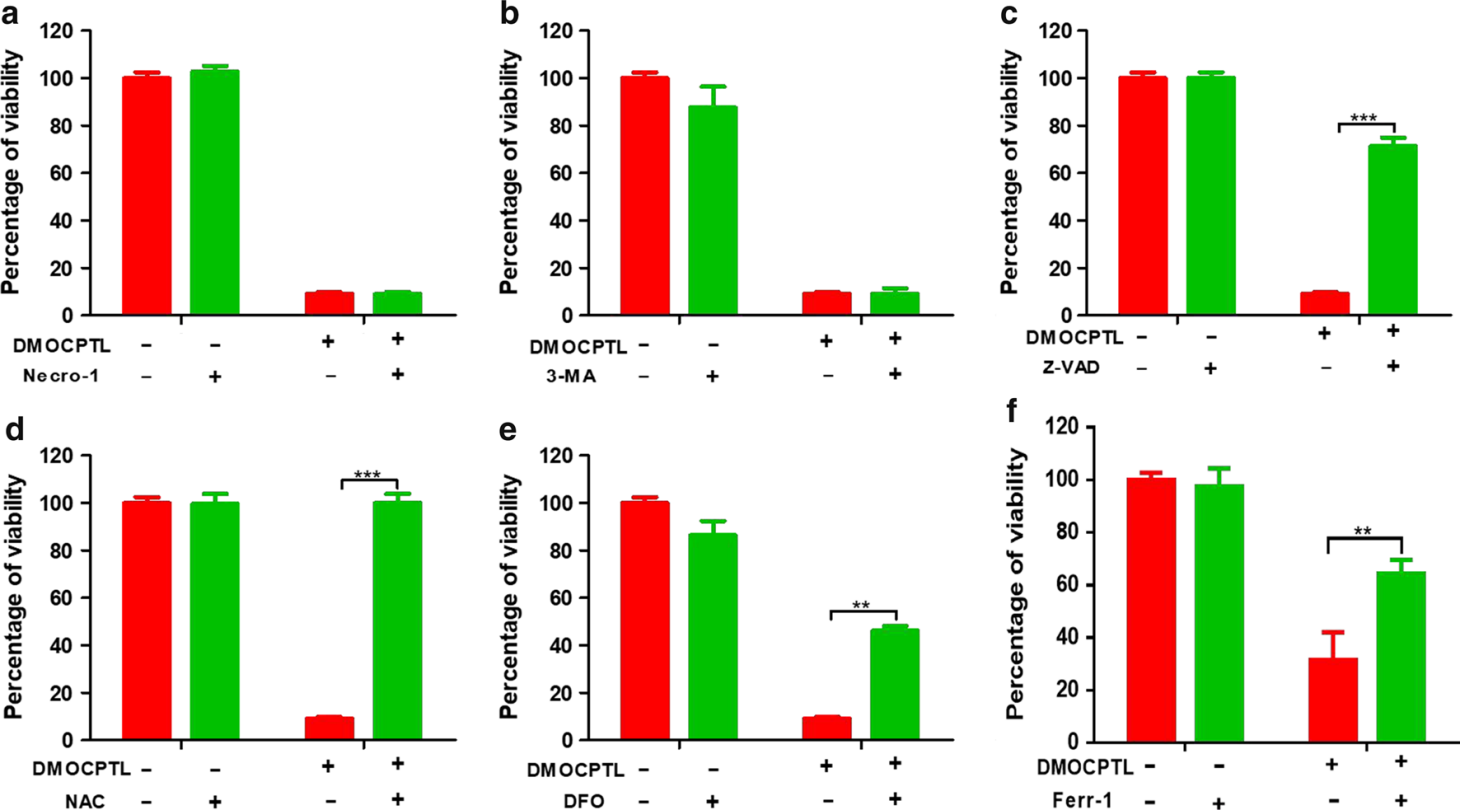
DMOCPTL induced apoptosis and ferroptosis of TNBC cells. **a** The percentage of cell viability after the combination of DMOCPTL (0.5 μM) with necrosis inhibitor necrostatin-1 (10 μM) for 72 h. **b** The percentage of cell viability after the combination of DMOCPTL (0.5 μM) with 3-methyladenine (5 mM) for 72 h. **c** The percentage of cell viability after the combination of DMOCPTL (0.5 μM) with Z-VAD-FMK (10 μM) for 72 h. **d** The percentage of cell viability after the combination of DMOCPTL (0.5 μM) with ferroptosis inhibitors *N*-Acetyl-L-cysteine (5 mM), **e** deferoxamine (200 μM), and **f** Ferrostatin-1 (10 μM). Analysis results represented mean ± SD, **P < 0.01, ***P < 0.001

To reveal the mechanism of **DMOCPTL**, the death manners were analyzed. In this experiment, the apoptosis inhibitor Z-VAD-FMK, cell autophagy inhibitor 3-methyladenine, cell necrosis inhibitor necrostatin-1, and ferroptosis inhibitors *N*-acetyl-L-cysteine, deferoxamine and Ferrostatin-1 were used. As shown in Fig. 3, there was no significant change in the percentage of cell viability after the combination of **DMOCPTL** and cell necrosis inhibitor necrostatin-1, which indicated that **DMOCPTL** could not induce cell necrosis (Fig. 3a). Meanwhile, the percentage of cell viability was not affected clearly after the combination of **DMOCPTL** and cell autophagy inhibitor 3-methyladenine, which suggested that **DMOCPTL** could not induce cell autophagy (Fig. 3b). We then investigated whether the effect of **DMOCPTL** was achieved by inducing cell apoptosis. The apoptosis inhibitor Z-VAD-FMK was combined with **DMOCPTL** for treatment of MDA-MB-231 cells for 48 h. The effect of **DMOCPTL** was inhibited by cell apoptosis inhibitor Z-VAD-FMK, which prompted us to infer that the effect of **DMOCPTL** may be through inducing cell apoptosis (Fig. 3c). As cell apoptosis inhibitor could not completely inhibit the effect of **DMOCPTL**, we further analyzed whether **DMOCPTL** could induce ferroptosis. The effect of **DMOCPTL** was inhibited by cell ferroptosis inhibitors *N*-Acetyl-L-cysteine, deferoxamine and Ferrostatin-1 which demonstrated that the effect of **DMOCPTL** was achieved partly by inducing cell ferroptosis (Fig. 3d–f). These results suggested that the effect of **DMOCPTL** may be achieved by inducing apoptosis and ferroptosis of TNBC cells.

**Fig. 4:**
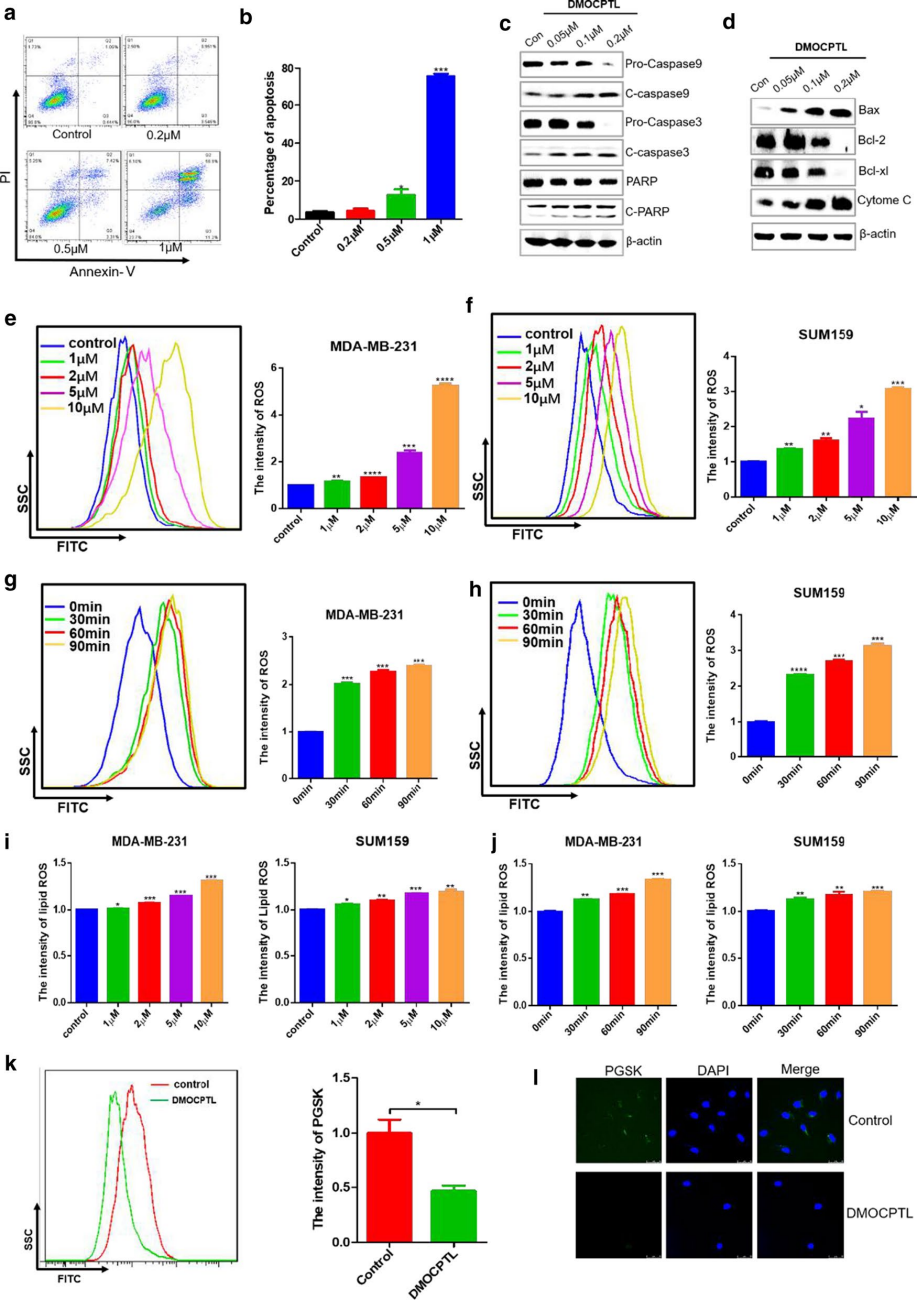
**DMOCPTL** induced apoptosis and ferroptosis of TNBC cells. **a** The representative images of cell apoptosis in MDA-MB-231 cells for 48 h at different concentrations of 0.2 μM, 0.5 μM and 1 μM, respectively. **b** The statistical results of cell apoptosis assays. **c** Western blot analysis of caspase 9, cleaved caspase 9, caspase 3, cleaved caspase 3, PARP, and cleaved PARP after treatment of **DMOCPTL** for 48 h in MDA-MB-231 cells at different concentrations of 0.05 μM, 0.1 μM and 0.2 μM, respectively. **d** Western blot analysis of apoptosis related proteins of mitochondrial pathway after the treatment of **DMOCPTL** for 48 h in MDA-MB-231 at different concentrations of 0.05 μM, 0.1 μM and 0.2 μM, respectively. **e** The representative images and statistical results of ROS after the treatment of **DMOCPTL** with a dose-dependent manner for 2 h in MDA-MB-231 cells. **f** The representative images and statistical results of ROS after the treatment of **DMOCPTL** with a dose-dependent manner for 2 h in SUM159 cells. **g** The representative images and statistical results of ROS after the treatment of **DMOCPTL** with a time-dependent manner in MDA-MB-231 cells. **h** The representative images and statistical results of ROS after the treatment of **DMOCPTL** with a time-dependent manner in SUM159 cells. **i** The statistical results of lipid ROS after the treatment of **DMOCPTL** with a dose-dependent manner for 2 h in MDA-MB-231 and SUM159 cells. **j** The statistical results of lipid ROS after the treatment of **DMOCPTL** with a time-dependent manner for 2 h in MDA-MB-231 and SUM159 cells. **k** The representative images and statistical results of intracellular Fe^2+^ by PGSK assay after the treatment of **DMOCPTL** for 48 h in MDA-MB-231 cell. **l** The fluorescence intensity of PGSK after the treatment of **DMOCPTL** for 48 h in MDA-MB-231 cells by immunofluorescence assay. Analysis results represented mean ± SD, *P < 0.05, **P < 0.01, ***P < 0.001

In order to investigate the effect of **DMOCPTL** on cell apoptosis, the cell apoptosis assay was performed by Annexin V/PI staining. The percentage of apoptosis cells was the sum of early apoptosis (Annexin V + /PI-) and late apoptosis (Annexin V + /PI +). As shown in Fig. 4b, the percentage of cell apoptosis was 3.60 ± 1.53, 4.27 ± 1.22, 12.60 ± 1.95 and 75.60 ± 1.30 after the treatment of **DMOCPTL** at the concentration of 0, 0.2 µM, 0.5 µM and 1 µM, respectively. Furthermore, the mechanism of **DMOCPTL** inducing apoptosis was studied by western blot assay. The relative levels of anti-apoptotic proteins Bcl-2 and Bcl-xl were decreased with a dose-dependent manner, while the relative level of apoptotic protein Bax was dramatically increased with a dose-dependent manner. Bax, Bcl-2 and Bcl-xl were important mitochondrial proteins which regulated the release of cytochrome C. Moreover, **DMOCPTL** could lead to the release of the cytochrome C and the cleavage of caspase 9, caspase 3 and PARP, which induced apoptosis of MDA-MB-231 cells. These results suggested that **DMOCPTL** could induce apoptosis of MDA-MB-231 cells by mitochondria pathway (Fig. 4c, d).

We then investigated the mechanism of **DMOCPTL** inducing cell ferroptosis. The accumulation of intracellular ROS was increased after the treatment of **DMOCPTL** in a time- and dose-dependent manner (Fig. 4e–h). The lipid ROS, a key characteristic of ferroptosis, was increased synchronously after the treatment of **DMOCPTL** in a time- and dose-dependent manner (Fig. 4i, j). Moreover, we detected the Fe^2+^ intensity, the results showed that Fe^2+^ intensity was significantly increased (PGSK intensity decreased) after the treatment of **DMOCPTL** for 48 h (Fig. 4k, l). These above investigations suggested that **DMOCPTL** could induce apoptosis and ferroptosis of TNBC cells.

### DMOCPTL reduced GPX4 protein level by inducing GPX4 ubiquitination

**Fig. 5:**
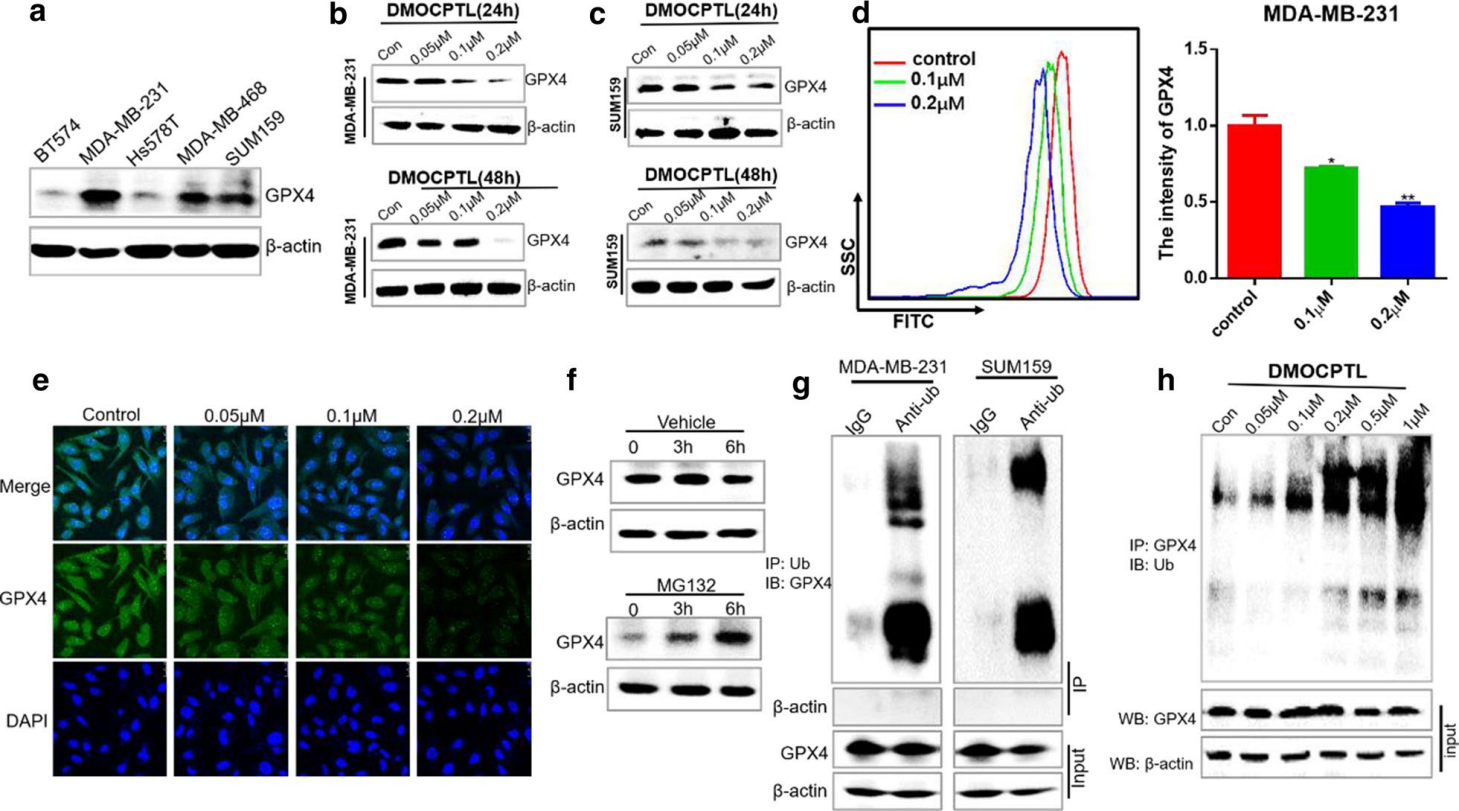
DMOCPTL regulated GPX4 protein level by inducing GPX4 ubiquitination. **a** Western blot analyzed GPX4 expression in five TNBC cell lines. **b** Western blot analyzed GPX4 expression in MDA-MB-231 cells and SUM159 cells **c** after the treatment of **DMOCPTL** for 24 or 48 h at different concentrations. **d** MDA-MB-231 cells with **DMOCPTL** treatment for 48 h were collected and incubated with GPX4 antibody and corresponding FITC-conjunct second antibody. The fluorescence intensity of GPX4 expression was analyzed by flow cytometry. **e** The fluorescence intensity of GPX4 after the treatment of **DMOCPTL** for 48 h in MDA-MB-231 cells by immunofluorescence assay. **f** The protein level GPX4 after treatment of proteasome inhibitor MG132 at 3 h and 6 h. **g** MDA-MB-231 and SUM159 cell lysates were incubated with IgG or anti-Ub antibody at 4 °C for 6 h, then agarose A + G was added, co-incubated at 4 °C overnight on rotary shaker. The samples were washed with PBS and analyzed by western blot assay. **h DMOCPTL** was added into MDA-MB-231 cells and incubated for 24 h. The proteins were collected and incubated GPX4 antibody for 2 h on ice, then agarose G was added and co-incubated at 4 °C overnight. The ubiquitination level of GPX4 was analyzed by western blot assay with anti-Ub antibody. Analysis results represented mean ± SD, *P < 0.05, **P < 0.01

It is well known that GPX4 was a significant negative regulator of ferroptosis [32, 33]. Western blot results showed that the protein level of GPX4 was highly expressed in MDA-MB-231, SUM159 and MDA-MB-468 cells (Fig. 5a). **DMOCPTL** could reduce GPX4 protein level in a dose-dependent manner in both MDA-MB-231 and SUM159 cells (Fig. 5b, c). Moreover, the flow cytometry assay and immunofluorescence assay also suggested that **DMOCPTL** down-regulated the level of GPX4 with a dose-dependent manner (Fig. 5d, e).

Ubiquitination is an enzymatic process that involves the binding of a ubiquitin protein to a substrate protein. The most common result of ubiquitination is the degradation of the protein via the proteasome. We rationalized that an alternative way to lead to cell death by inducing ferroptosis would be through inducing ubiquitination of GPX4. We treated MDA-MB-231 cells with proteasome inhibitor MG132 and the result showed that GPX4 was accumulated with the treatment of MG132. This result suggested that GPX4 was modified by ubiquitination and the degradation of GPX4 in MDA-MB-231 occurs via the ubiquitin–proteasome system (Fig. 5f). Co-immunoprecipitation analysis with anti-Ubiquitin antibody also indicated that GPX4 was ubiquitinated in MDA-MB-231 and SUM159 cells (Fig. 5g). Therefore, the ubiquitination of GPX4 after the treatment of **DMOCPTL** was analyzed. The result indicated that **DMOCPTL** could increase the ubiquitination of GPX4 with a dose-dependent manner (Fig. 5h). These data demonstrated that **DMOCPTL** could reduce GPX4 level by inducing ubiquitination of GPX4.

### DMOCPTL induced ubiquitination by directly binding to GPX4

**Fig. 6:**
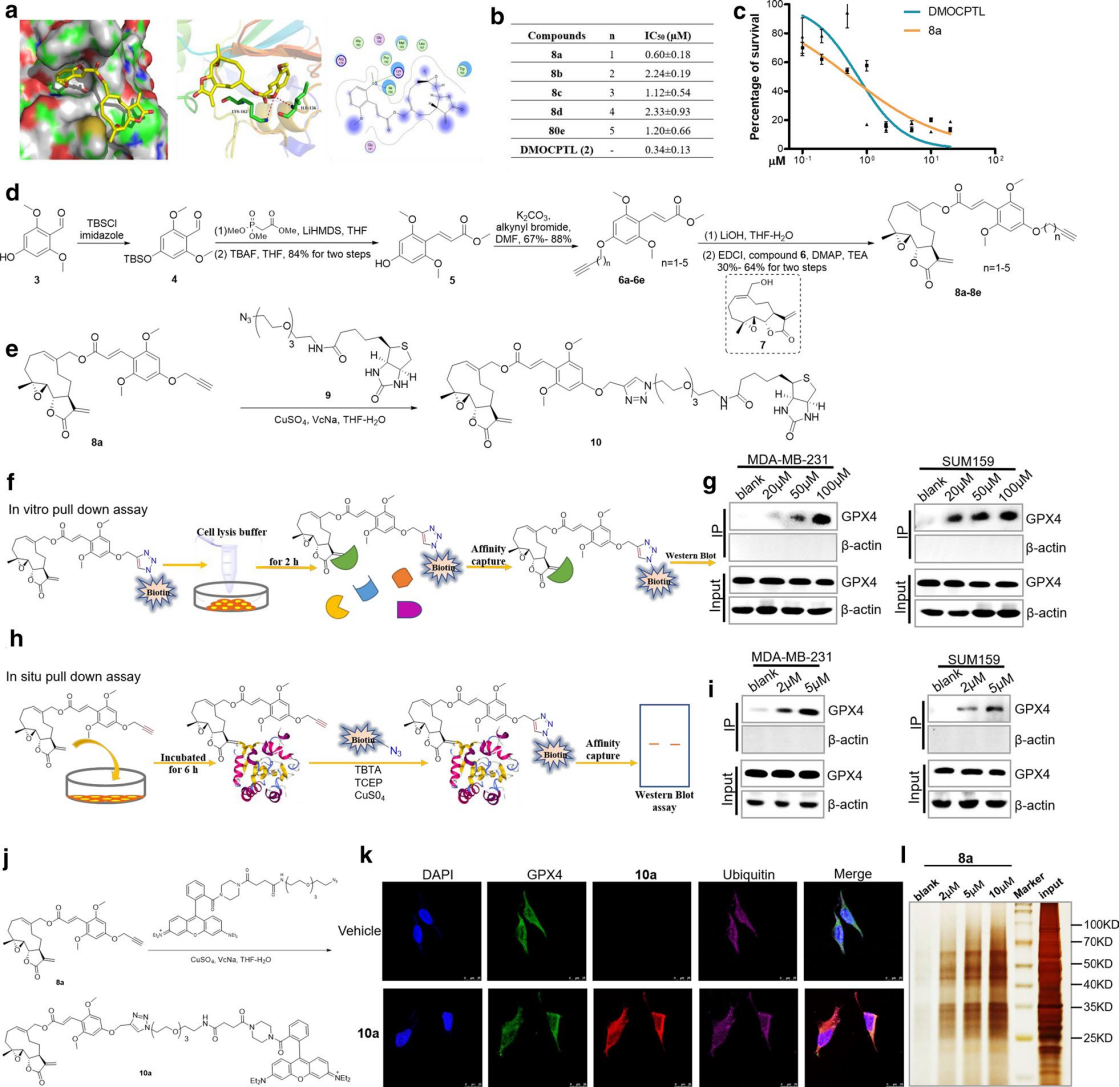
DMOCPTL regulated ubiquitination by directly binding to GPX4. **a** Docking of **DMOCPTL** into the active site of GPX4 (PDB code: 5H5S). The protein structure is shown as a cartoon diagram with selected residues labeled (left). Surface representation of the protein residues (Middle). 2D representation of binding interaction of **DMOCPTL** and GPX4 (right). **b** The IC50 values of **DMOCPTL** probes in MDA-MB-231 cells after the treatment for 72 h by MTT assay. **c** The effect of **DMOCPTL** and probe **8a** on the viability of MDA-MB-231 cells after treatment for 72 h. **d** The scheme for synthesis of probes **8a–8e**. **e** The scheme for synthesis of probe **10**. **f** The workflow of probe **10** for identifying **DMOCPTL**-interacting proteins by in vitro pull-down assay in MDA-MB-231 and SUM159 cells. **g** MDA-MB-231 and SUM159 cell lysates were incubated with probe **10** for 2 h at room temperature. After incubated with streptavidin conjunct agarose A + G beads, the bead-bound proteins were collected and detected by western blot experiments. **h** The workflow of probe **8a** for identifying DMOCPTL-interacting proteins by in situ pull-down assay in MDA-MB-231 and SUM159 cells. **i** MDA-MB-231 and SUM159 cells after the treatment of probe **8a** for 6 h were collected and performed click reaction to conjugate biotin with probe **8a**. After incubated with streptavidin conjunct agarose A + G beads, the bead-bound proteins were collected and detected by western blot experiments. **j** The scheme for synthesis of probe **10a**. **k** The co-localization of GPX4 (green), Ubiquitin (purple), probe **10a** (red); the nucleus DAPI (blue) in MDA-MB-231 cells by immunofluorescence assay. **l** MDA-MB-231 cells after the treatment of probe **8a** for 6 h were collected and performed click reaction to conjugate biotin with probe **8a**. After incubated with streptavidin conjunct agarose A + G beads, the bead-bound proteins were collected and analyzed by sliver staining

To further get understanding of the mechanism of **DMOCPTL** reducing level of GPX4, interaction of **DMOCPTL** with GPX4 was studied. **DMOCPTL** was docked with the GPX4 (PDB code: 5H5S). As shown the optimal pose of **DMOCPTL** oriented the styryl ring into the hydrophobic pocket, forming strong hydrophobic interactions with Trp163 and Pro182. Meanwhile, the oxygen atom of methoxyl group formed hydrogen bond with the side chain of Lys 162 (3.41 Å) and Ile156 (3.17 Å) (Fig. 6a). **DMOCPTL** was tightly bound into the active site of GPX4, and the target binding site is consistent with Sakamoto’s result [34]. The docking of **DMOCPTL** with GPX4 protein suggested that **DMOCPTL** may directly interact with GPX4. To further analyze the interaction of **DMOCPTL** with GPX4, we synthesized a series of **DMOCPTL** probes (Fig. 6d, e). The anti-TNBC activity showed that probe **8a** showed a comparable IC_50_ value with that of **DMOCPTL** (Fig. 6b, c). Then, the in vitro pull-down experiment showed that **DMOCPTL** could bind with GPX4 directly in MDA-MB-231 and SUM159 cells (Fig. 6f, g). To better imitate the intracellular environment and membrane permeability of **DMOCPTL**, in situ pull-down assay was performed. The results also indicated that **DMOCPTL** could bind GPX4 directly in MDA-MB-231 and SUM159 cells (Fig. 6h, i). To further visualize the interaction of GPX4 and **DMOCPTL**, we synthesized a fluorescent probe **10a** (Fig. 6j). The confocal microscopy results showed that **DMOCPTL** colocalized with GPX4 and ubiquitin (Fig. 6k). To further reveal the E3 enzymes involved in GPX4 ubiquitination induced by **DMOCPTL**, in situ pull down assay with probe **8a** was performed (Fig. 6l).

### GPX4 was overexpressed in breast cancer

**Fig. 7:**
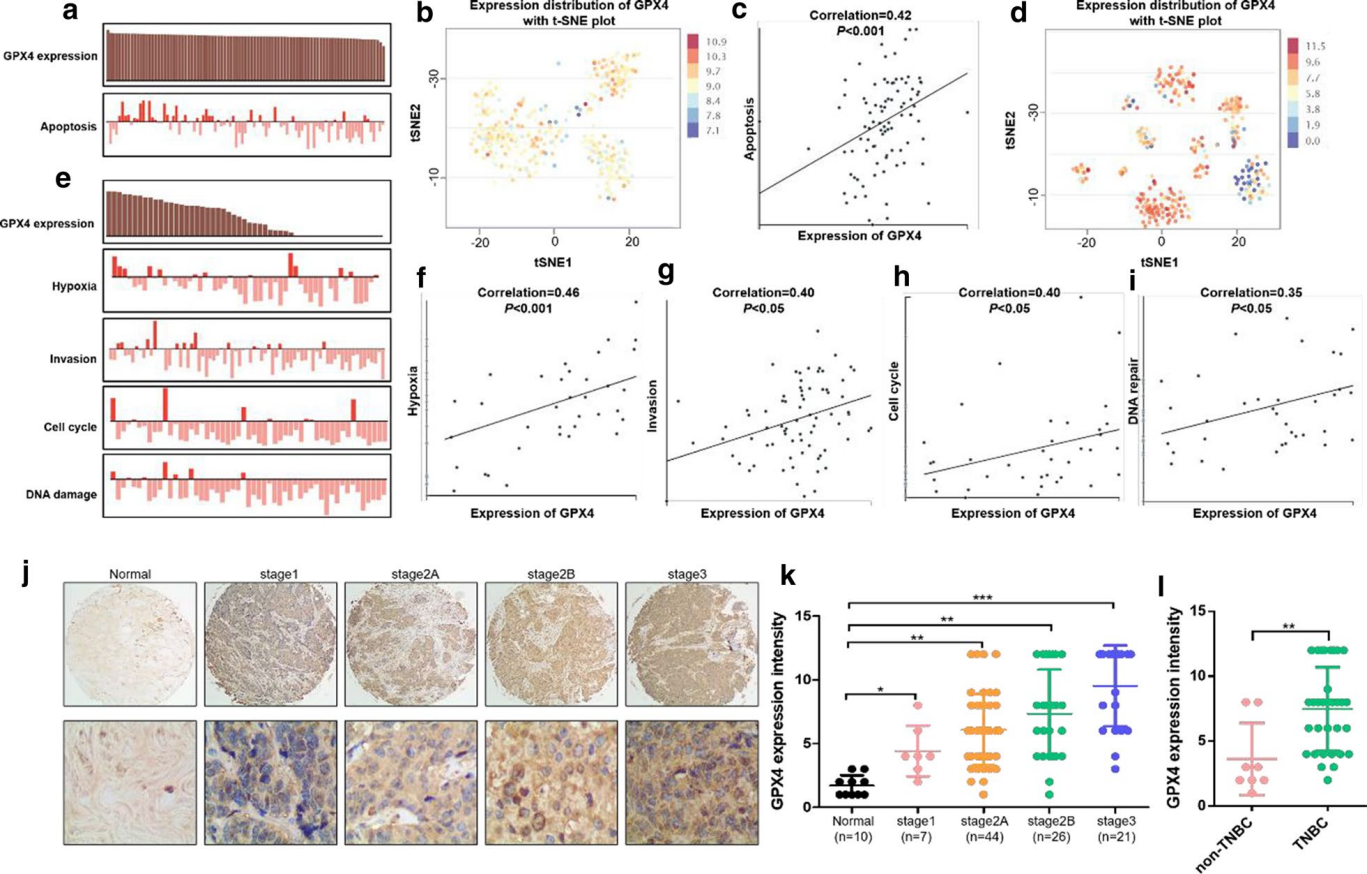
GPX4 was overexpressed in breast cancer. **a** The Functional state analysis of GPX4 in breast cancer cells in patient 1. **b** The expression distribution of GPX4 in breast cancer cells in CancerSEA database in patient 1. Every point represents a single cell. **c** The correlation of GPX4 with cell apoptosis was analyzed in patient 1. **d** The expression distribution of GPX4 in breast cancer cells in CancerSEA database in patient 2. Every point represents a single cell. **e** The Functional state analysis of GPX4 in breast cancer cells in patient 2. The correlation of GPX4 with hypoxia (**f**), invasion (**g**), cell cycle (**h**) and DNA damage (**I**) were investigated in breast cancer cells in CancerSEA database in patient 2. Every point represents a single cell. **j** IHC analysis of GPX4 expression in breast cancer tissue with different stages and normal breast tissue. **k** The statistical results of GPX4 expression in breast cancer tissue with different stages and normal breast tissue. **l** The IHC results of GPX4 expression in TNBC and non-TNBC breast cancer tissues. Analysis results represented mean ± SD, *P < 0.05, **P < 0.01, ***P < 0.001

GPX4 expression in breast cancer was still little investigated. To analyze GPX4 expression in breast cancer, we investigated the related functional states of GPX4 in CancerSEA (http://biocc.hrbmu.edu.cn/CancerSEA/). GPX4 expression distribution with t-SNE showed that breast cancer cells with high GPX4 expression tended to cluster together in two patients (Fig. 7b, d), which suggests that GPX4 may promote the malignant progression of breast cancer. Furthermore, apoptosis, hypoxia, invasion, cell cycle and DNA damage were significantly related to GPX4 expression in breast cancer cells (Fig. 7a, c, e, f, g, h, i). The IHC assay showed that GPX4 protein was significantly increased in breast cancer tissue compared with normal breast tissue and related with breast cancer stages, which indicated the prominent role of GPX4 in breast cancer (Fig. 7j, k). Moreover, the level of GPX4 was higher in TNBC than non-TNBC in clinical samples, which suggested that GPX4 was very significant for TNBC (Fig. 7l).

### GPX4 knockdown induced ferroptosis and apoptosis of TNBC cells

**Fig. 8:**
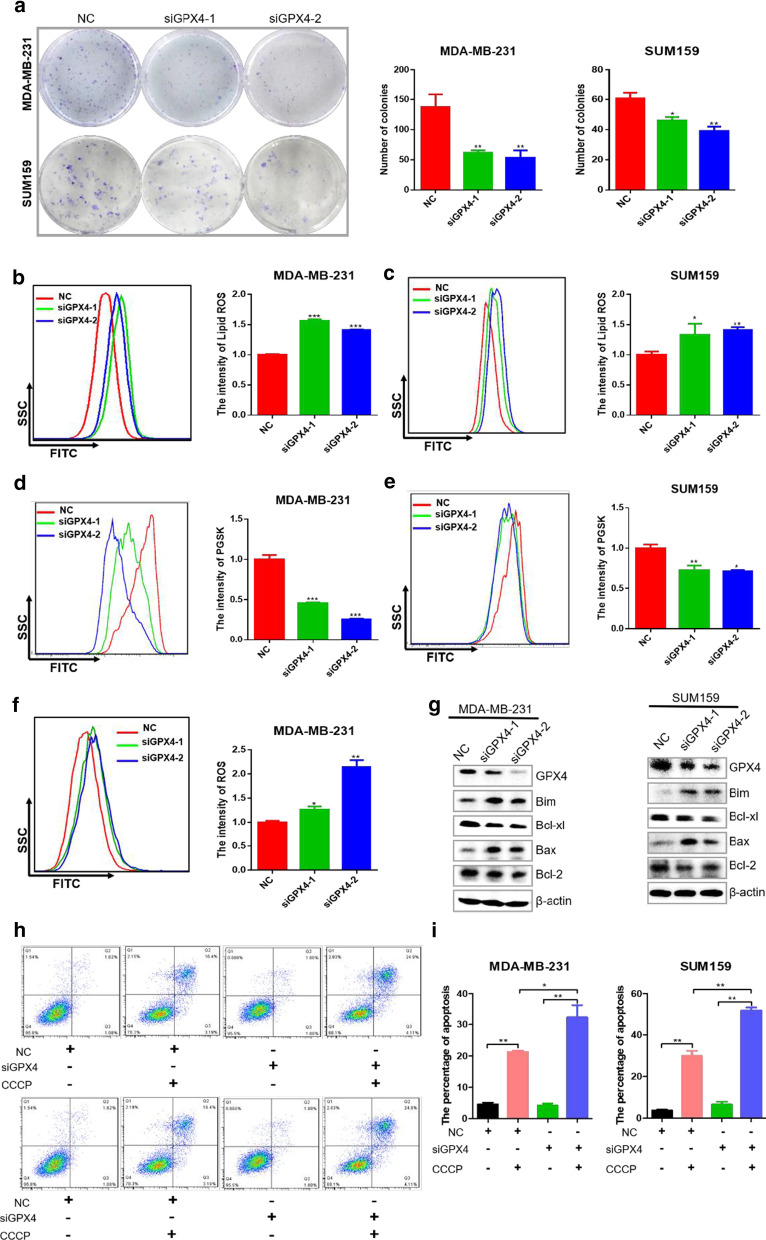
GPX4 knockdown induced cell ferroptosis and apoptosis of breast cancer cells. **a** The representative pictures and statistical results of colony formation in MDA-MB-231 and SUM159 cells after GPX4 knockdown. **b** MDA-MB-231 cells were transfected with GPX4 siRNA for 48 h, and then cells were collected and incubated with BODIPY for 1 h to detect the lipid ROS level by flow cytometry. The representative images (left) and statistical results (right) of lipid ROS after GPX4 knockdown. **c** The representative images (left) and statistical results (right) of lipid ROS after GPX4 knockdown in SUM159 cells by flow cytometry assay. **d** MDA-MB-231 cells were transfected with GPX4 siRNA for 48 h, and then cells were collected and incubated with PGSK for 1 h to detect intracellular Fe^2+^ level by flow cytometry. The representative images (left) and statistical results (right) of intracellular Fe^2+^ after GPX4knock down in MDA-MB-231 cells by PGSK assay. **e** The representative images (left) and statistical results (right) of intracellular Fe^2+^ after GPX4 knock down in SUM159 cells by flow cytometry assay. **f** MDA-MB-231 cells were transfected with GPX4 siRNA for 48 h, and then cells were collected and incubated with DCF-DA for 1 h to detect ROS level by flow cytometry. The representative images (left) and statistical results (right) of ROS after GPX4 knockdown. **g** Western blot analysis of apoptosis related proteins of mitochondrial pathway after GPX4 knockdown. **h** The representative images of cell apoptosis in MDA-MB-231 and SUM159 cells after GPX4 knockdown with the treatment of CCCP. **i** The statistical results of cell apoptosis in MDA-MB-231 and SUM159 cells after GPX4 knockdown with the treatment of CCCP. Analysis results represented mean ± SD, *P < 0.05, **P < 0.01, ***P < 0.001

To further investigated the role of GPX4 in TNBC cells, GPX4 protein was knocked down by siRNA. The number of colonies was clearly decreased after GPX4 knockdown in MDA-MB-231 and SUM159 cells (Fig. 8a). Moreover, knockdown of GPX4 by siRNA increased the accumulation of lipid ROS (Fig. 8b, c), the intensity of intracellular Fe^2+^ (Fig. 8d, e), and intracellular ROS (Fig. 8f).These results demonstrated that GPX4 regulated ferroptosis in TNBC cells.

It was reported that knockdown of GPX4 induced apoptosis of glioma cells [32]. Accordingly, we speculated that GPX4 knockdown might induce apoptosis of breast cancer cells. To verify our speculation, we evaluated apoptosis related proteins by western blot assay. The results showed that the anti-apoptotic proteins Bcl-2 and Bcl-xl of GPX4-siRNA group were decreased, while the apoptotic protein Bax and Bim were dramatically increased compared with the control-siRNA group (Fig. 8g). Furthermore, the percentage of apoptosis induced by CCCP (an apoptosis inducer) was significantly increased after GPX4 knockdown (Fig. 8h, i). These results showed that GPX4 regulated ferroptosis and apoptosis in TNBC cells.

### GPX4 induced apoptosis through up-regulation of EGR1 in TNBC cells

**Fig. 9:**
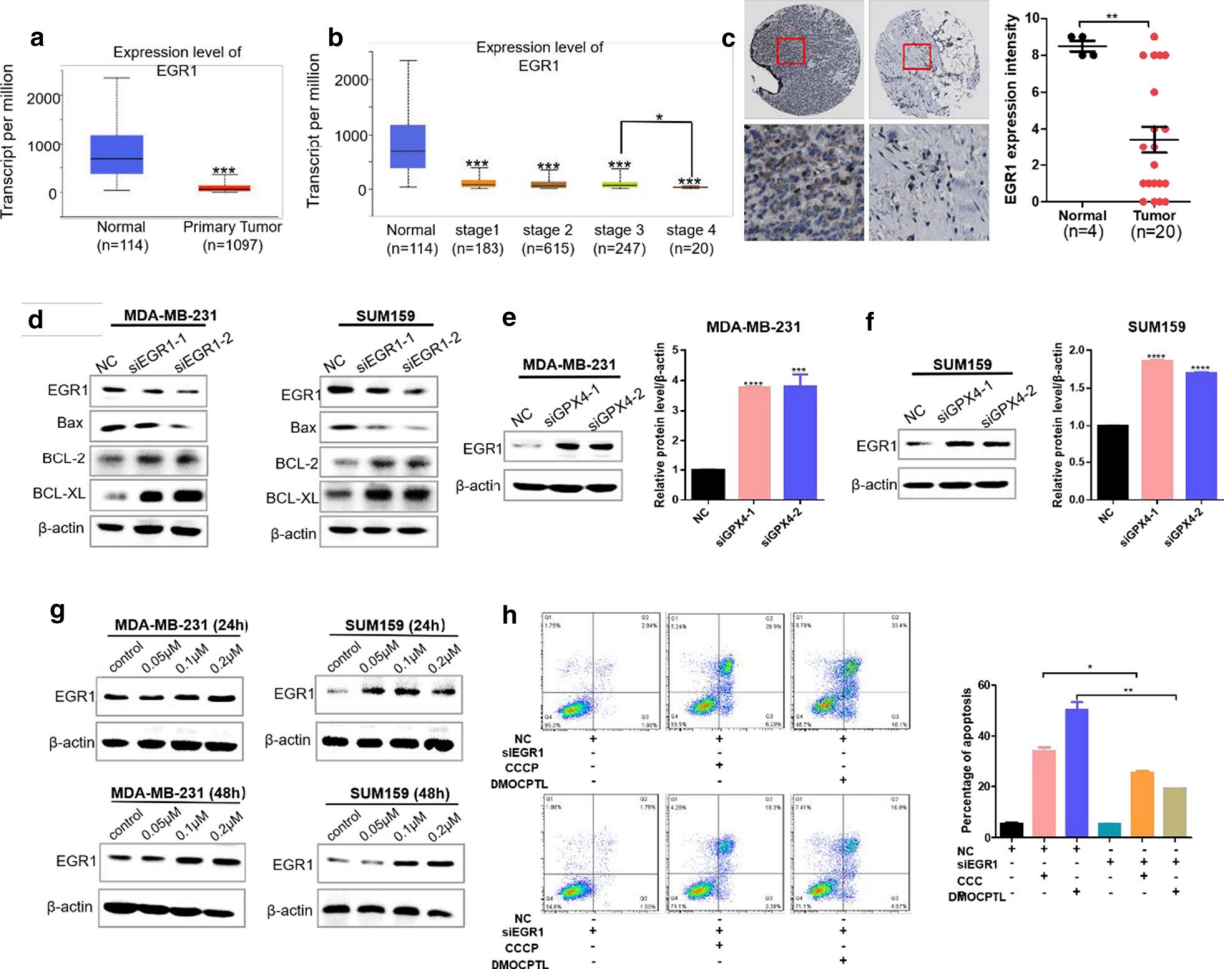
GPX4 regulated apoptosis through upregulation of EGR1 in TNBC cells. **a** The expression analysis of EGR1 in normal individuals and breast cancer tissues by UALCAN. **b** The expression analysis of EGR1 in breast cancer tissue with different stages and normal breast tissue by UALCAN. **c** The IHC analysis of EGR1 expression in breast cancer tissue and normal breast tissue in the human protein atlas datasets. **d** Western blot analysis of apoptosis related proteins of mitochondrial pathway after EGR1 knockdown. **e** Western blot analysis of EGR1 expression after GPX4 knockdown in MDA-MB-231 cells. **f** Western blot analysis of EGR1 expression after GPX4 knockdown in SUM159 cells. **g** Western blot analysis of EGR1 expression after DMOCPTL treatment for 24 h or 48 h in MDA-MB-231 cells (left) and SUM159 cells (right). **h** The representative images of cell apoptosis in MDA-MB-231 cells after EGR1 knockdown with the treatment of CCCP or DMOCPTL (left). The statistical results of cell apoptosis in MDA-MB-231 cells after EGR1 knockdown with the treatment of CCCP or DMOCPTL (right). Analysis results represented mean ± SD, *P < 0.05, **P < 0.01, ***P < 0.001

Early growth response-1 (EGR1) is an early response gene that is involved in growth, differentiation, apoptosis, neurite outgrowth, and wound healing [35]. As reported, EGR1 was closely related to cell apoptosis and ROS production. Moreover, ROS production induced EGR1 expression to induce cell apoptosis. GPX4 was a key regulator of ferroptosis by induced ROS especially lipid ROS production. Accordingly, we rationally speculated that GPX4 would induce EGR1 expression and cell apoptosis. We investigated the gene transcription level in breast cancer cells using the UALCAN database (http://ualcan.path.uab.edu/index.html). EGR1 was significantly decreased in breast cancer tissues compared to normal breast tissues (Fig. 9a), and EGR1 gene expression was negative correlation with breast cancer stages (Fig. 9b). Moreover, the IHC assay showed that the EGR1 protein expression in breast cancer tissue was clearly decreased compared with that in normal breast tissue (Fig. 9c). To verify the effect of EGR1 in mitochondria-mediated apoptosis of TNBC cells, western blot assay was performed. The relative levels of anti-apoptotic proteins Bcl-2 and Bcl-xL were increased, while the relative level of apoptotic protein Bax was dramatically decreased after EGR1 knockdown (Fig. 9d). Furthermore, EGR1 protein was significantly increased after GPX4 knockdown (Fig. 9e, f). **DMOCPTL** clearly increased EGR1 expression both in MDA-MB-231 and SUM159 cells with a dose dependent manner (Fig. 9g). Moreover, the percentage of apoptosis induced by CCCP (an apoptosis inducer) and **DMOCPTL** were significantly decreased after EGR1 knockdown (Fig. 9h), which indicated that EGR1 inhibited cell apoptosis and the apoptosis inducing effect of **DMOCPTL** was rescued by EGR1 knockdown.

### DMOCPTL could inhibit growth of TNBC and prolong survival life of mice in vivo and had no obvious toxicity

**Fig. 10:**
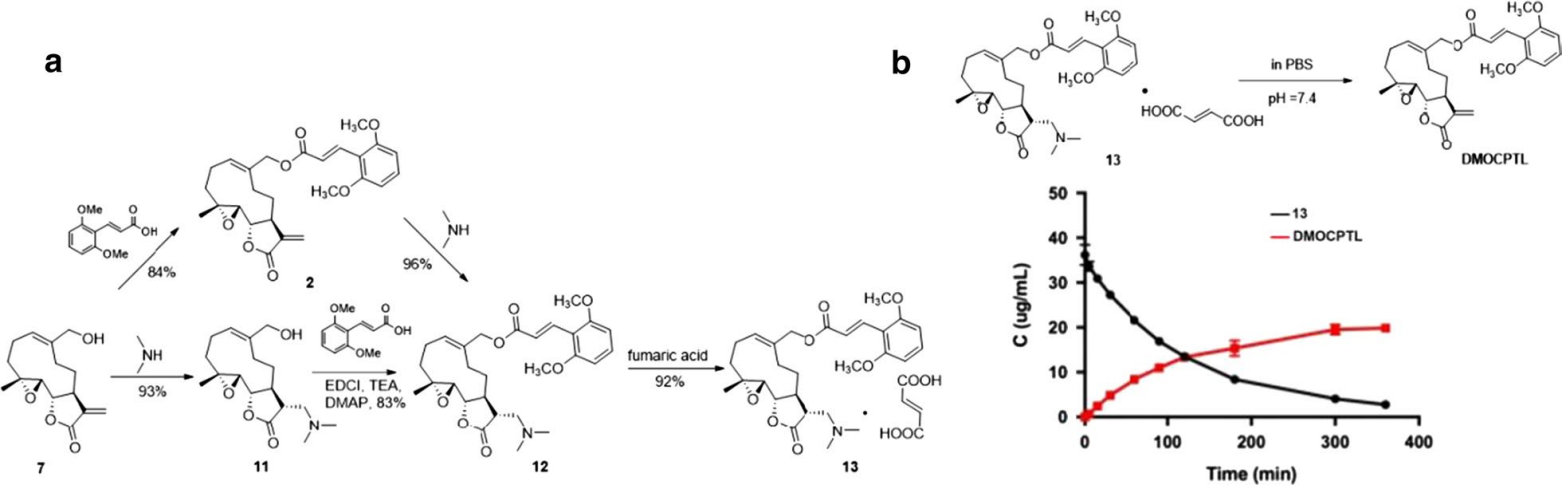
The prodrug of **DMOCPTL. a** Schematic diagram for synthesis of compound **13**, a prodrug of **DMOCPTL**. **b** Concentration–time curve of compound **13** and **DMOCPTL** in PBS (pH = 7.4)

Taking account of significant anti-TNBC activity of **DMOCPTL** in vitro and its novel mechanism inducing mitochondria-mediated apoptosis and ferroptosis through ubiquitination of GPX4, we planned to evaluate its potency to inhibit TNBC in vivo and investigate its effect on GPX4 and apoptosis related proteins in vivo. However, **DMOCPTL** showed low solubility in water. In order to improve the water solubility of **DMOCPTL**, we designed to convert **DMOCPTL** into a prodrug **13**. Michael-type addition of **7** with dimethylamine gave alcohol **11**, subsequent EDCI/DMAP–mediated coupling with 2,6-dimethoxycinnamic acid produced ester **12**. Alternatively, direct Michael addition of **2** selectively occurred on *γ*-butyrolactone moiety produced **12** without influence of the cinnamyl moiety. Finally, **12** and equivalent fumaric acid in methanol gave its salt **13** (Fig. 10a). Compound **13** could gradually release **DMOCPTL** in PBS solution (pH = 7.4) (Fig. 10b).

**Fig. 11:**
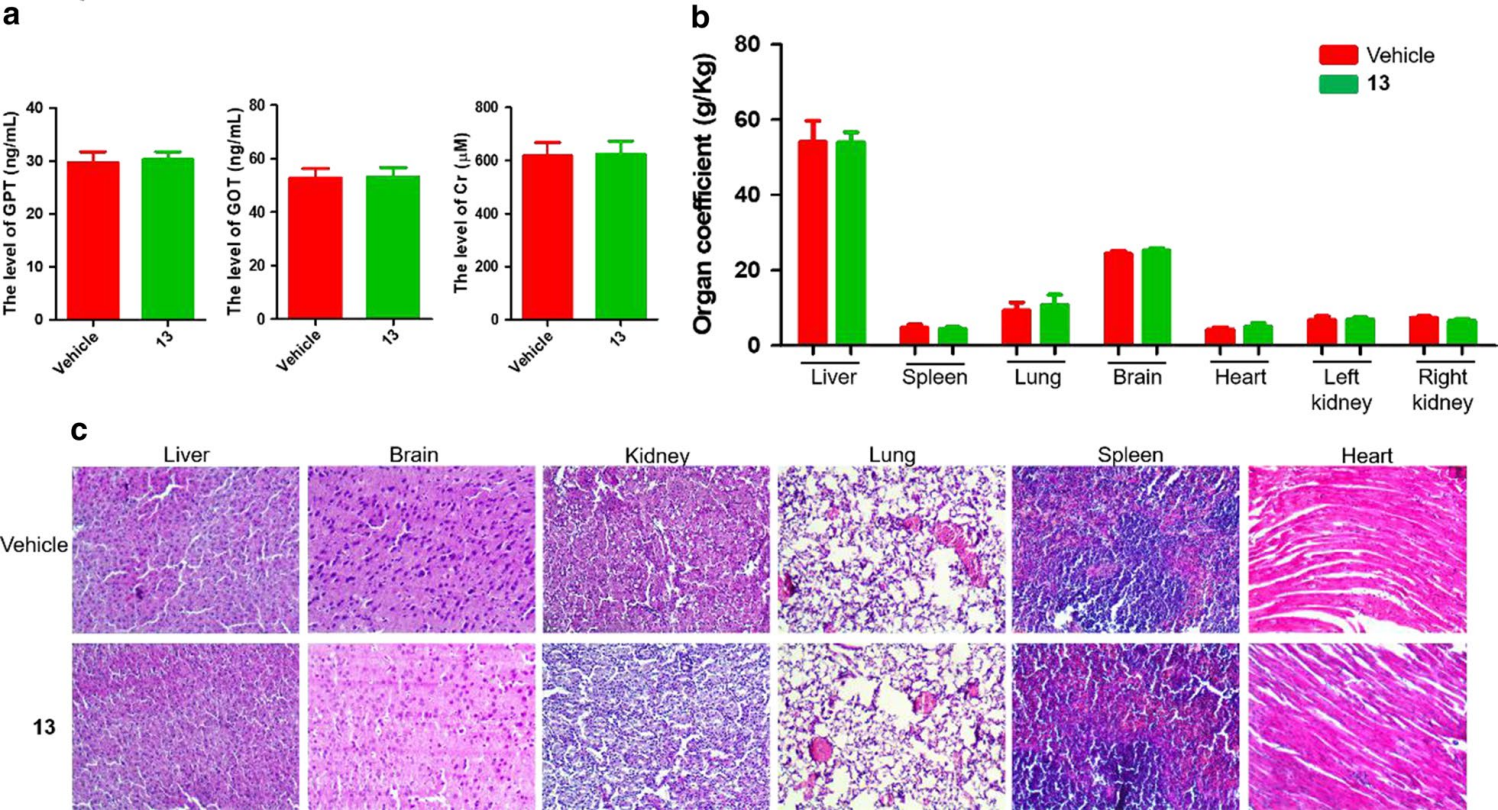
The toxicity study of compound **13** by oral administration at a dose of 500 mg/kg. **a** The level of GPT, GOT and Cr in serum after oral administration of **13** for 72 h in Bar b/c mice. **b** The organ coefficient of liver, spleen, lung, brain, heart and kidney were calculated. The organ coefficient was calculated by organ weight divided by body weight. **c** The histomorphology of liver, spleen, brain, kidney, lung, spleen and heart were analyzed by HE staining

To explore the safety of **13**, compound **13** was administrated orally at a dose of 500 mg/kg. The level of GPT, GOT and Cr in serum was detected, and no obvious change was observed comparing with that of vehicle control group (Fig. 11a). The weight and histomorphology of the major organs including liver, spleen, lung, kidney, heart, and brain in **13**-group showed no obvious change compared with vehicle control group (Fig. 11b, c).

**Fig. 12:**
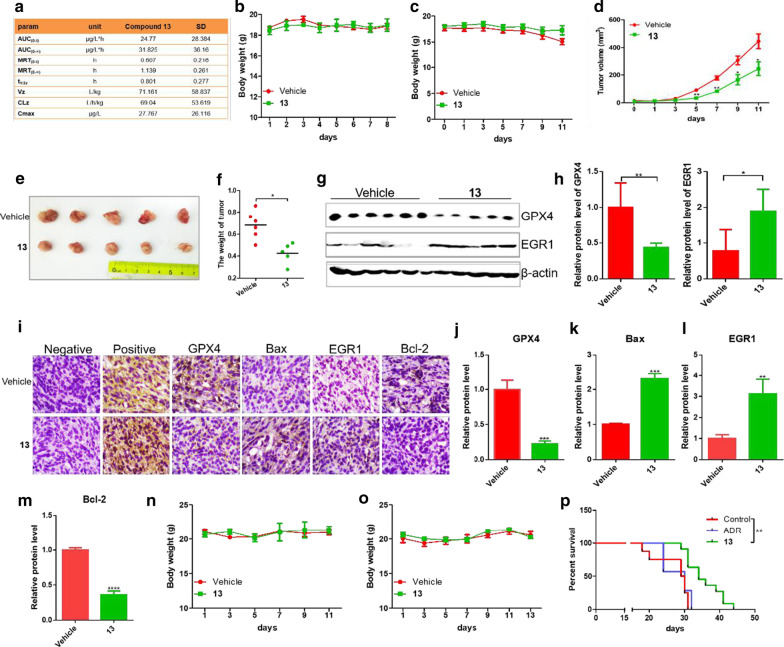
**DMOCPTL** could inhibit TNBC and prolong survival life of mice in vivo and had no obvious toxicity. **a** Compound **13** was injected to SD rats by intravenous administration at a dose of 1 mg/kg. Then, the blood sampling from jugular sinus of rats were collected at 2 min, 5mins, 15mins, 30mins, 1 h, 2 h, 3 h, 4 h, 6 h and 8 h after administration. The samples were treated and analyzed by LC/MS. The pharmacodynamics of **13** on SD rats was calculated by DAS 3.3. **b** The body weight of mice after intravenous administration of **13** at a dose of 50 mg/kg compared with vehicle group. **c** The body weight of mice after treatment of **13** at a dose of 7.5 mg/kg compared with vehicle group. **d** The statistical results of tumor volume after treatment of **13** at a dose of 7.5 mg/kg. **e** The picture of tumors after administration of **13** compared with vehicle group. **f** The weight of tumors after the treatment of compound **13** compared with vehicle group. **g** The levels of GPX4 and EGR1 protein expression in tumors by western blot assay. **h** The statistical results of GPX4 and EGR1 protein expression in tumors. **i** The IHC assay of GPX4, EGR1, and mitochondria-mediated apoptosis related proteins Bax and Bcl-2 in tumors. PBS instead of primary antibody was used as negative control. Breast cancer marker MUC16 was used as positive control. **j** The statistical results of GPX4, Bax (**k**), EGR1 (**l**) and Bcl-2 (**m**) by IHC assay after **13** treatment. **n** The body weight of mice after oral administration of **13** at a dose of 500 mg/kg. **o** The body weight of mice during treatment of **13** at a dose of 50 mg/kg every other day compared with vehicle group. **p** The statistical results of survival life after treatment of **13** at a dose of 50 mg/kg and clinically used drug ADR as a positive control by intraperitoneal administration at a dose of 2 mg/kg every 2 days. Analysis results represented mean ± SD, *P < 0.05, **P < 0.01

The pharmacodynamics of **13** was also investigated, and the results are shown in Fig. 12a. To further identify the safety of **13**, acute toxicity assay was performed. Bar b/c mice were treated with **13** at a dose of 50 mg/kg by intravenous injection or vehicle control for 7 days. No obvious toxic reaction and loss of body weight was observed after treatment of **13** (Fig. 12b). To determine the effect of compound **13** in vivo, orthotopic breast cancer mouse model was used. The 4T1 cells were implanted into the mammary fat pads of Bar b/c mice. After treatment of **13**, the tumor sizes of mice and their body weights were monitored and recorded every 2 days. The results showed that compound **13** significantly inhibited the tumor growth in vivo with no apparent loss of body weight compared with control group at the dose of 7.5 mg/kg (Figs. 12c–e). The tumor weight was also significantly reduced after treatment of compound **13** compared with control group (Fig. 12f). The protein level of GPX4 in tumor was decreased significantly in compound **13**-treated group compared with vehicle group (Fig. 12g, h). The relative level of EGR1 was dramatically increased in compound **13**-treated group compared with vehicle group (Fig. 12g, h). The tumors were harvested for IHC staining. The IHC implied that **13** treatment markedly down-regulated the expression levels of GPX4 and Bcl-2, and up-regulated the expression levels of EGR1 and Bax (Fig. 12i–m).

Furthermore, we planned to study the efficacy of **13** by oral administration. Prior to the study of the anti-breast cancer efficacy in vivo, an acute toxicity study of **13** by oral administration was conducted in Bar b/c mice. A single oral administration of **13** at a dose of 500 mg/kg was administered to the mice. These mice were observed for 14 days. During the study period, all mice remained alive, and no significant side effect was observed. The body weight of Bar b/c mice was not changed obviously (Fig. 12n). These results demonstrated that oral administration of **13** was well-tolerated in Bar b/c mice at a dose of 500 mg/kg in course of treatment and during the post-treatment period. After the oral administration of **13** with 50 mg/kg for 6 times every other day, no apparent change of the body weight was observed in comparison with vehicle group (Fig. 12o). The overall survival assay showed that compound **13** could significantly prolong the life span compared with control group. These results indicated that compound **13** could prolong survival life of mice in vivo and had no obvious toxicity (Fig. 12p).

The above results indicated that compound **13** was efficacious in inhibiting the growth of breast tumor and prolonging of survival span of mice in vivo and no obvious toxicity was observed.

## Discussion

TNBC is the most malignant breast cancer among various breast cancer subtypes. Its prognosis is far from satisfactory. TNBC remains a very challenging disease. There is an urgent and unmet need to discover effective drugs with novel mechanism for treatment of TNBC [36]. Induction of ferroptosis is a new effective strategy for treatment of TNBC [19–23]. GPX4 is an essential regulator of ferroptotic cell death [29]. Small molecule ferroptosis inducers could be summarized into two classes: one directly acts on mitochondrial VDAC2/3, inhibits cystine uptake by system Xc^−^ and ultimately results in lipid ROS accumulation in an NADH-dependent manner, including Erastin and RSL5, the other directly inhibits GPX4 activity without decreasing GSH, including RSL3 [2]. In contrast to these mechanisms, it was reported that FINO2 could initiate ferroptosis through GPX4 inactivation and iron oxidation [37] and FIN56 induced ferroptosis by degrading GPX4 with requirement of the enzymatic activity of acetyl CoA carboxylase [38].

Here, we identify a derivative of PTL, **DMOCPTL**, as a potential anti-TNBC agent, which can induce ferroptosis and apoptosis through ubiquitination of GPX4. This is the first report of inducing ferroptosis through ubiquitination of GPX4 by binding to GPX4 directly. **DMOCPTL** could significantly inhibit the proliferation of TNBC cells and the inhibitory activity was evidently potent than its mother compound PTL. GPX4 played prominent role in breast cancer and was significantly increased in breast cancer tissue compared with normal breast tissue and related with breast stages. Moreover, the expression of GPX4 in TNBC was higher than non-TNBC, which indicated the significance of GPX4 in TNBC cells. The mechanism study suggested that **DMOCPTL** bound to GPX4 leading to ubiquitination of GPX4, which induced ferroptosis and EGR1-mediated apoptosis of TNBC cells. Previous study had indicated that the loss of GPX4 expression could induce apoptosis [33]. Moreover, Lu et al.’s study demonstrated that GPX4 knockdown could induce apoptosis of glioma cells [32]. However, the mechanism for GPX4 regulation of apoptosis is still obscure. Here, we firstly reveal that GPX4 regulated apoptosis through up-regulation of EGR1 in TNBC cells (Figs. 8 and 9).

Acute toxicity study suggested that compound **13**, the prodrug of **DMOCPTL**, showed no obvious toxicity by intravenous administration and oral administration at a dose of 50 mg/kg and 500 mg/kg, respectively. Furthermore, in vivo experiment indicated that compound **13** effectively inhibited the growth of breast tumor and prolonged the life span of mice in vivo, and no obvious toxicity was observed during the experiment. The level of GPX4 was reduced, and EGR1 was increased in tumor for **13**-treated group compared with vehicle group.

## Conclusions

**Fig. 13:**
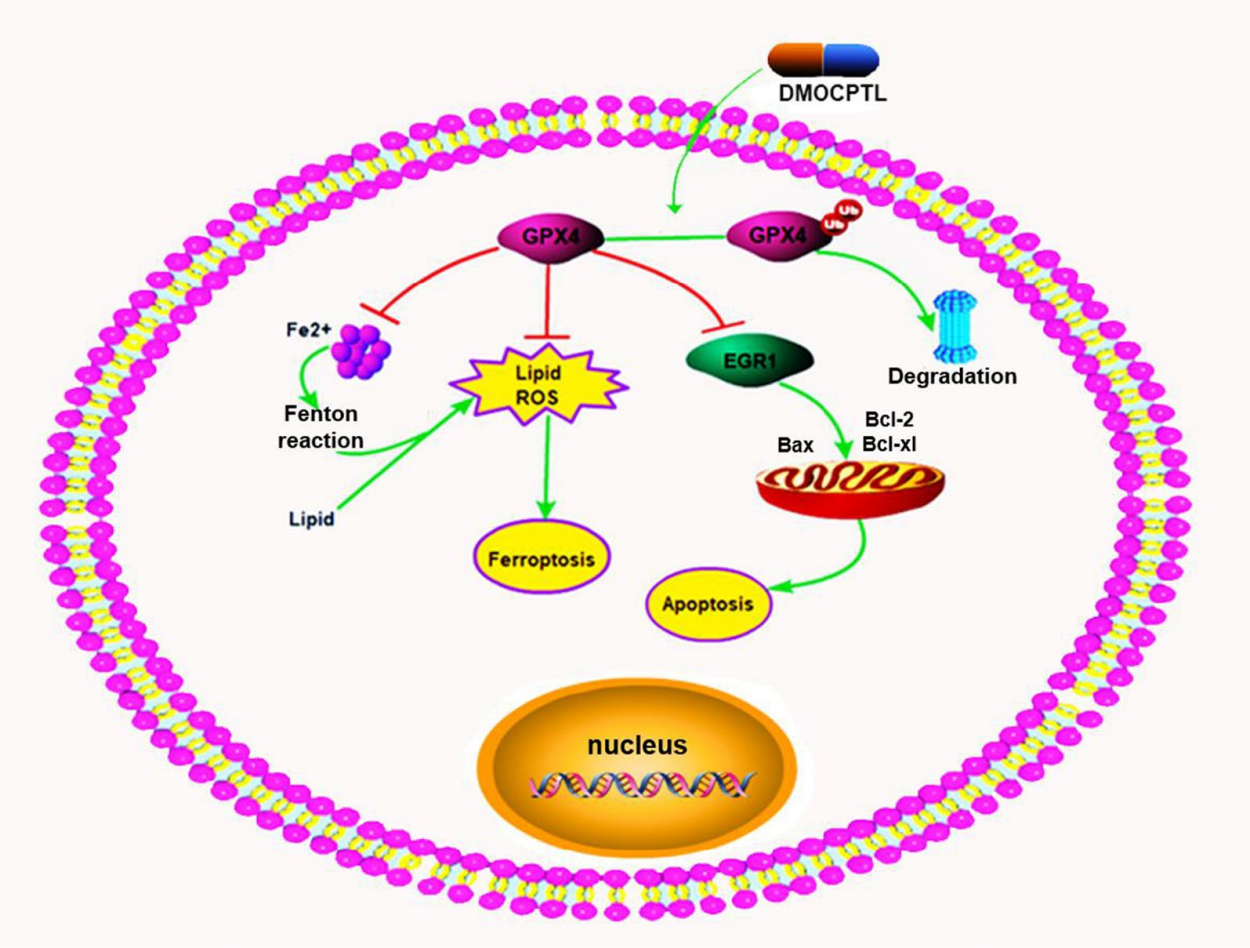
Schematic diagram of our proposed model of mechanism for how small molecule **DMOCPTL** induced ferroptosis and apoptosis of TNBC cells

In summary, these findings indicated that **DMOCPTL** could induce ferroptosis and EGR1-mediated apoptosis of TNBC cells through ubiquitination of GPX4 (Fig. 13). This is the first report of inducing ferroptosis through ubiquitination of GPX4. Moreover, our results link GPX4 to EGR1 leading to apoptosis of TNBC cells. On basis of these investigations, we propose that compound **13** deserves further studies as a promising lead compound for discovery of novel anti-TNBC drug.

## Supplementary Information

**Additional file 1**. NMR copies of compounds.

## Data Availability

All data generated or analyzed during this study are included either in this article or in Additional file 1.

## Abbreviations

PVL: Periventricular leukomalacia
DLBCL: Diffuse large B cell lymphomas
TNBC: Triple-negative breast cancer
ER: Estrogen receptors
PR: Progesterone receptors
HER-2: Human epidermal growth factor receptor 2
PTL: Parthenolide
AML: Acute myeloid leukemia
MTT: Thiazolyl blue tetrazolium bromide
