# Supplementary material for: Identification of a small molecule as inducer of ferroptosis and apoptosis through ubiquitination of GPX4 in triple negative breast cancer cells
Source: J Hematol Oncol. 2021 Jan 20;14:19. doi: 10.1186/s13045-020-01016-8 (PMC7816340; doi:10.1186/s13045-020-01016-8)

**Supplementary data for**

**Identification of a Small Molecule as Inducer of Ferroptosis and Apoptosis through Ubiquitination of GPX4 in Triple Negative Breast Cancer Cells**

Yahui Ding^a^, Xiaoping Chen^a^, Can Liu^a^, Weizhi Ge^a^, Qin Wang^a^, Xin Hao^a^, Mengmeng Wang^b^, Yue Chen*^,a^ and Quan Zhang*^,a^

*^a^State Key Laboratory of Medicinal Chemical Biology, College of Pharmacy and Tianjin Key Laboratory of Molecular Drug Research, Nankai University, Haihe Education Park, 38 Tongyan Road, Tianjin 300353, People’s Republic of China*

*^b^Accendatech Company, Ltd., Tianjin 300384, People's Republic of China*

1. **NMR copies of compounds………………………………S2**
2. **NMR copies of compounds**


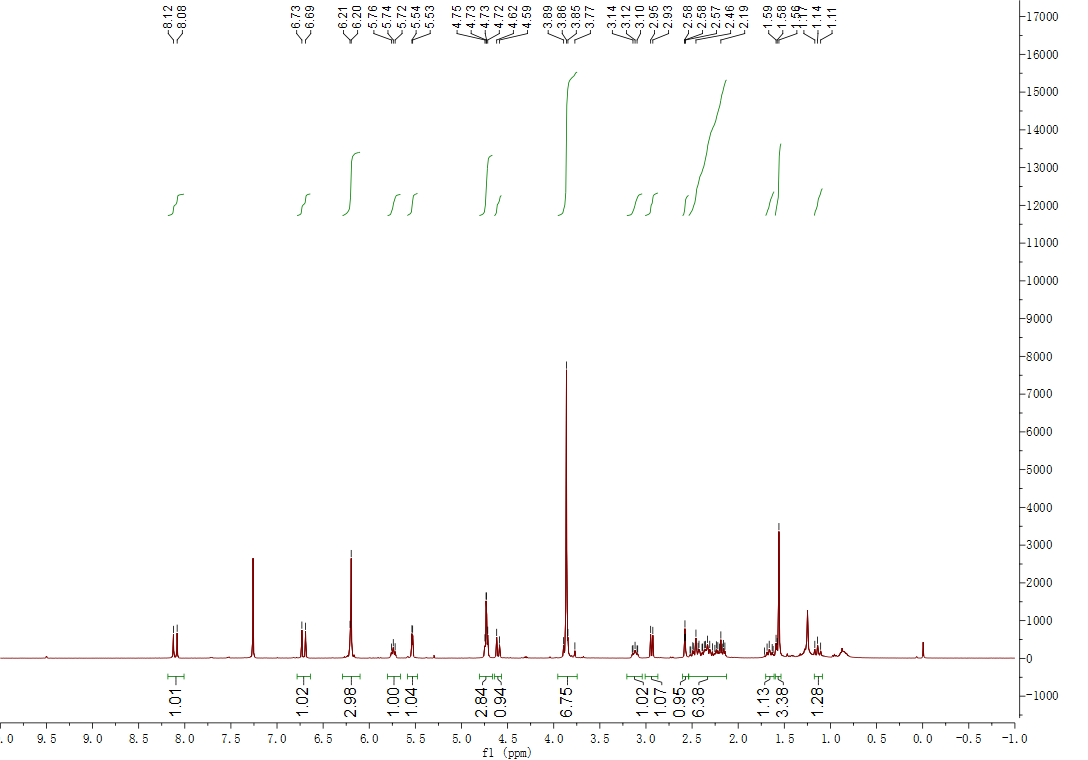


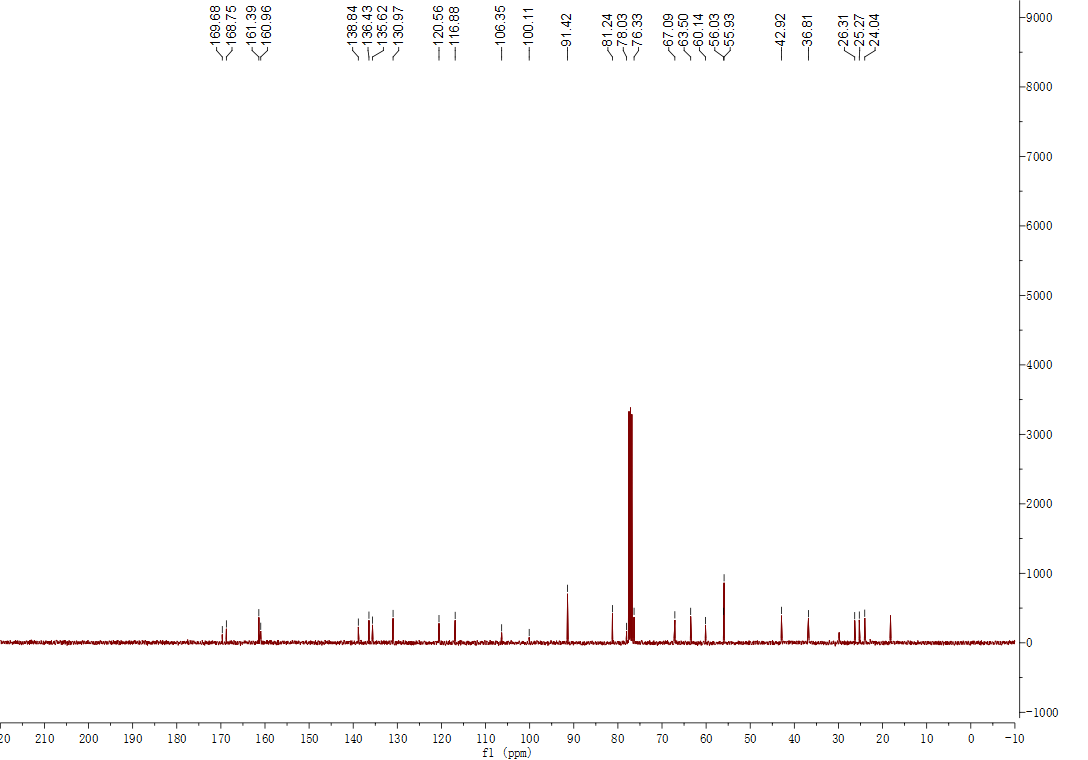


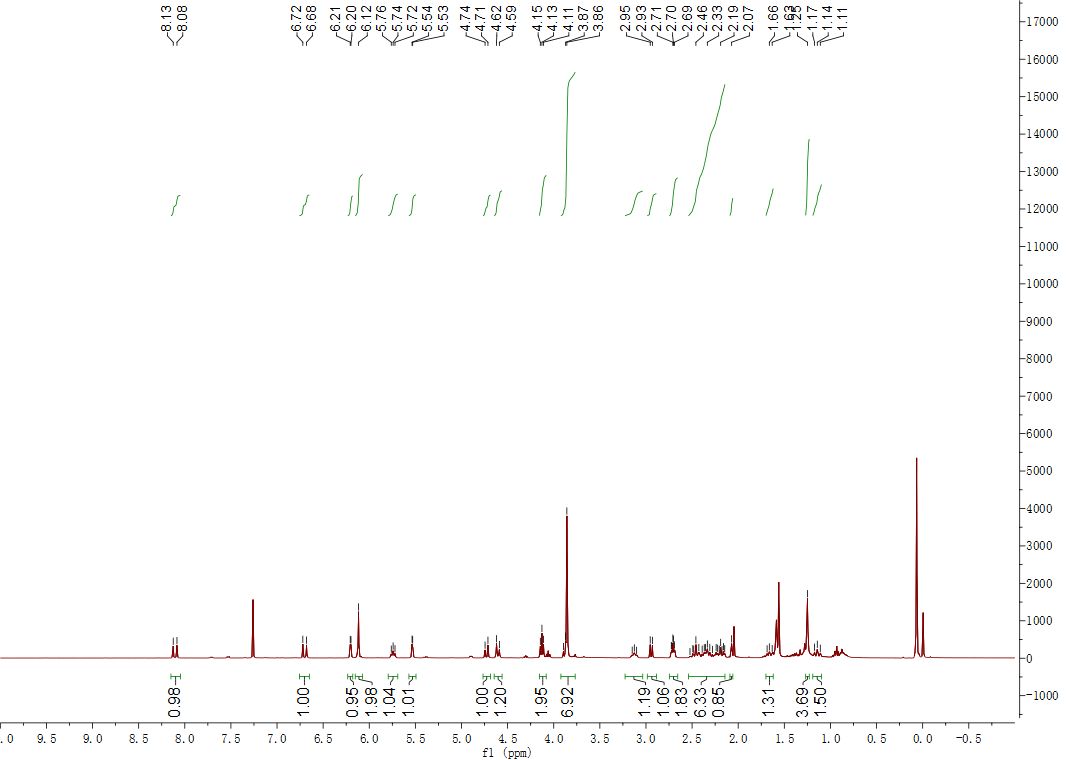


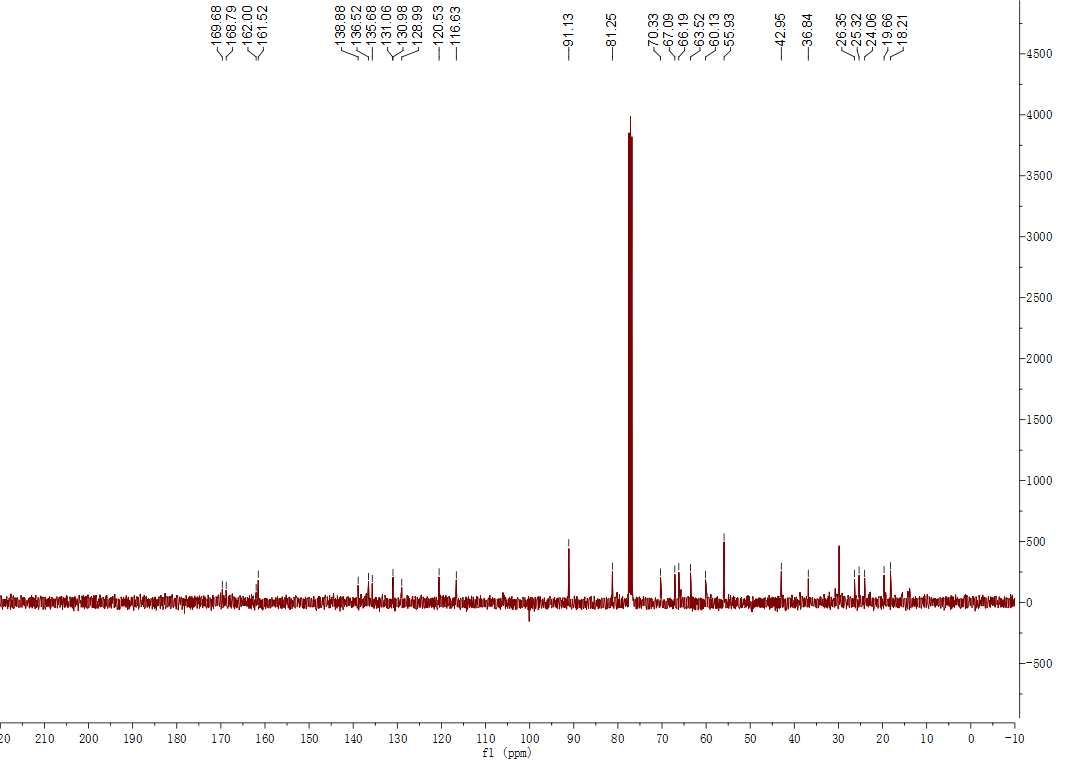


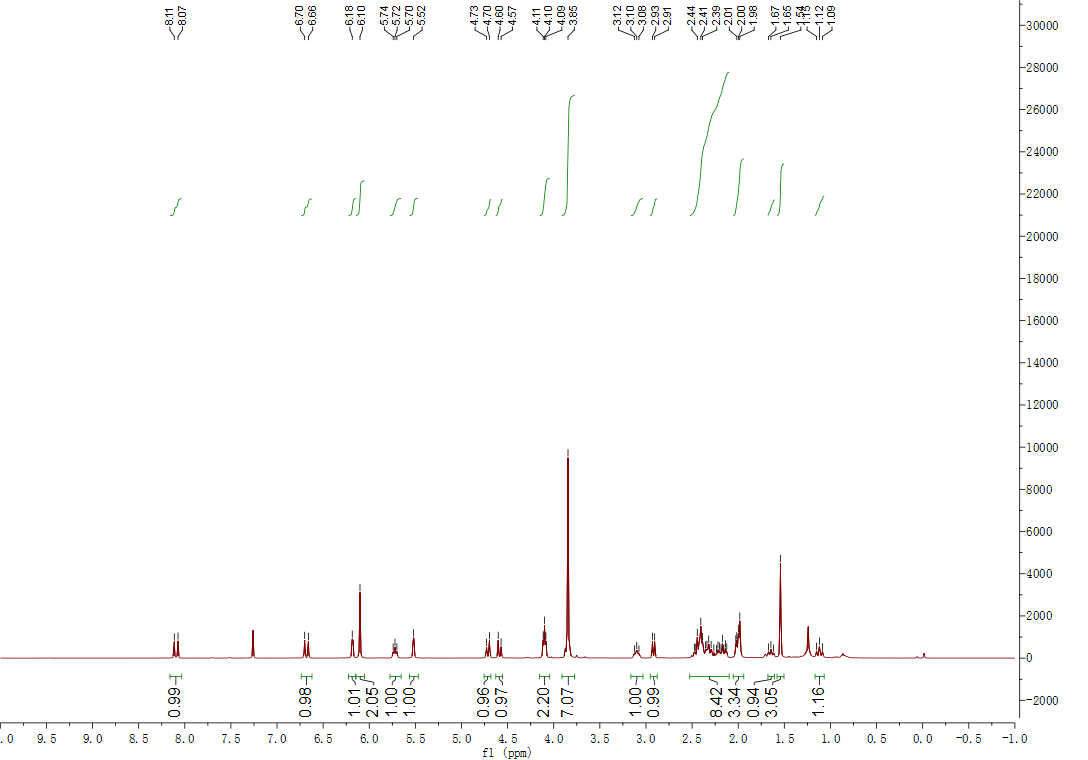


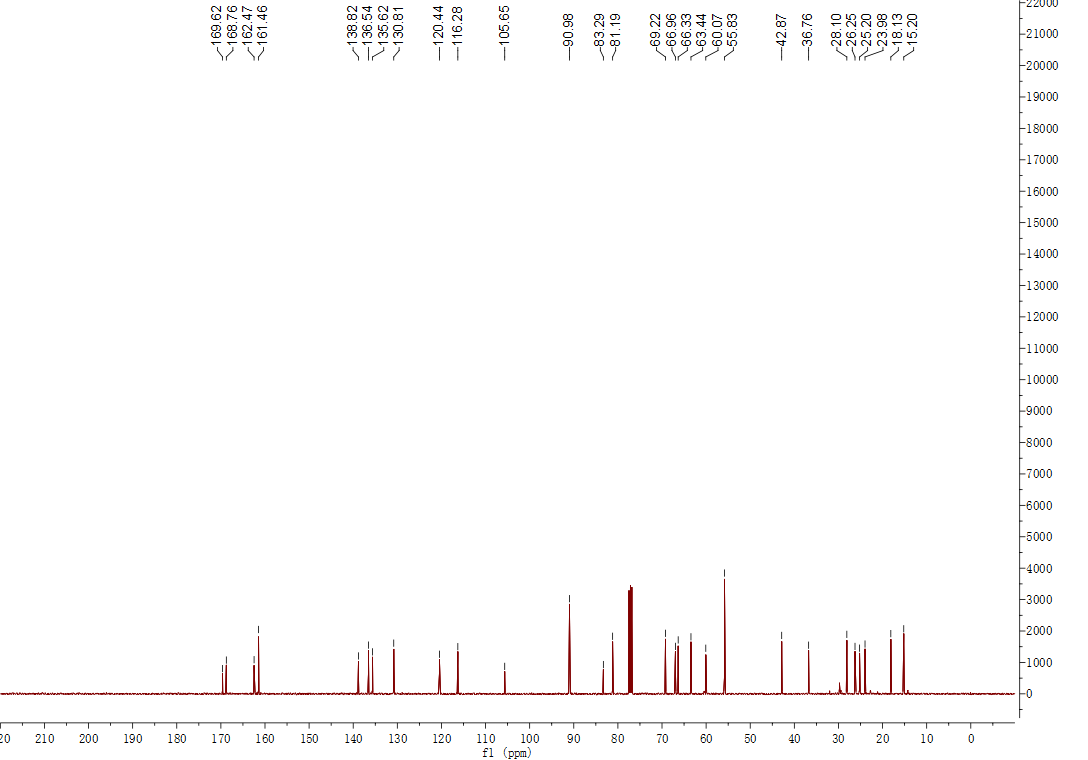


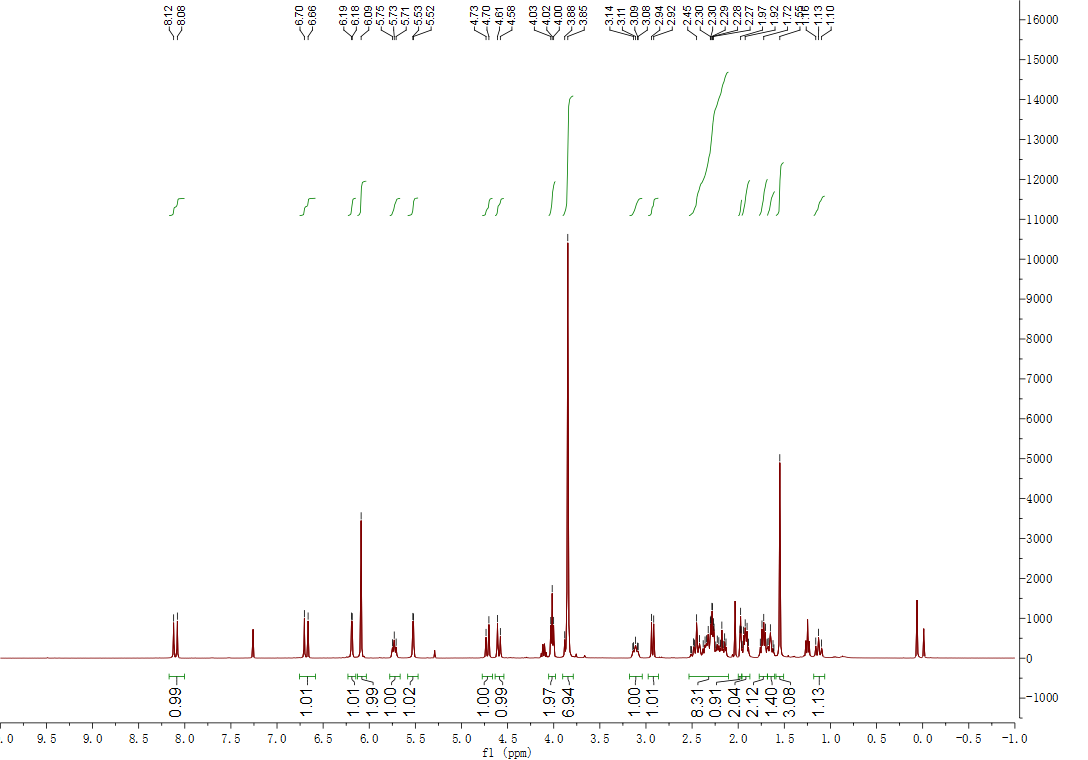


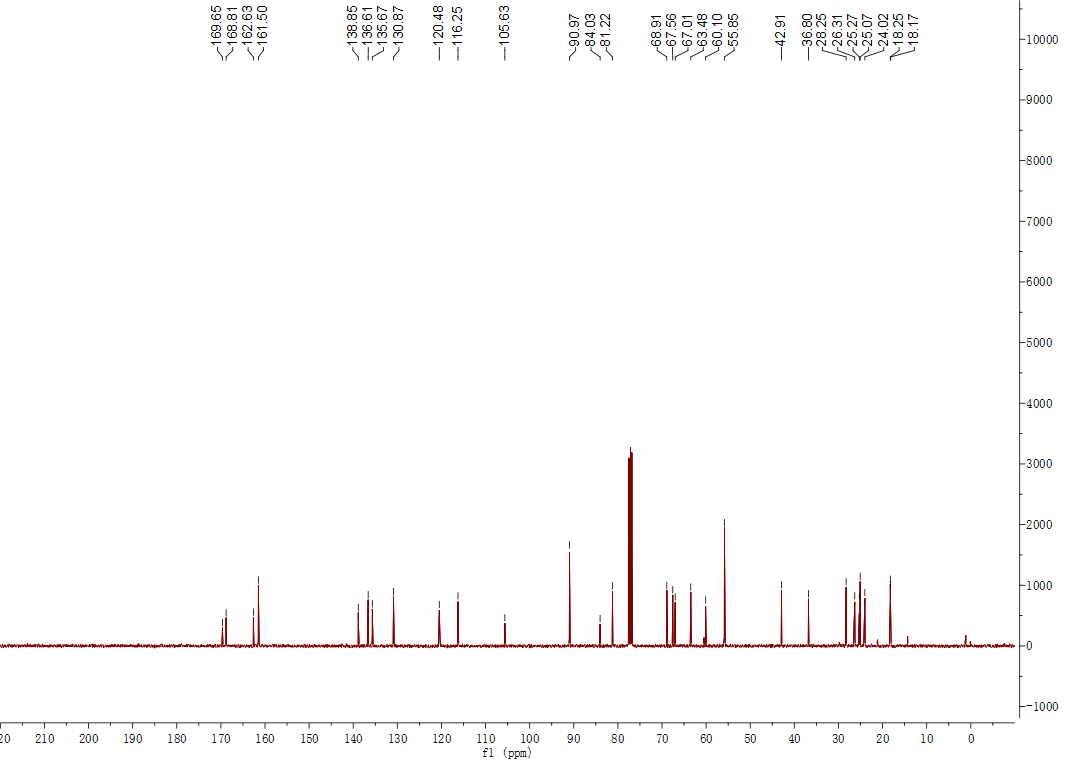


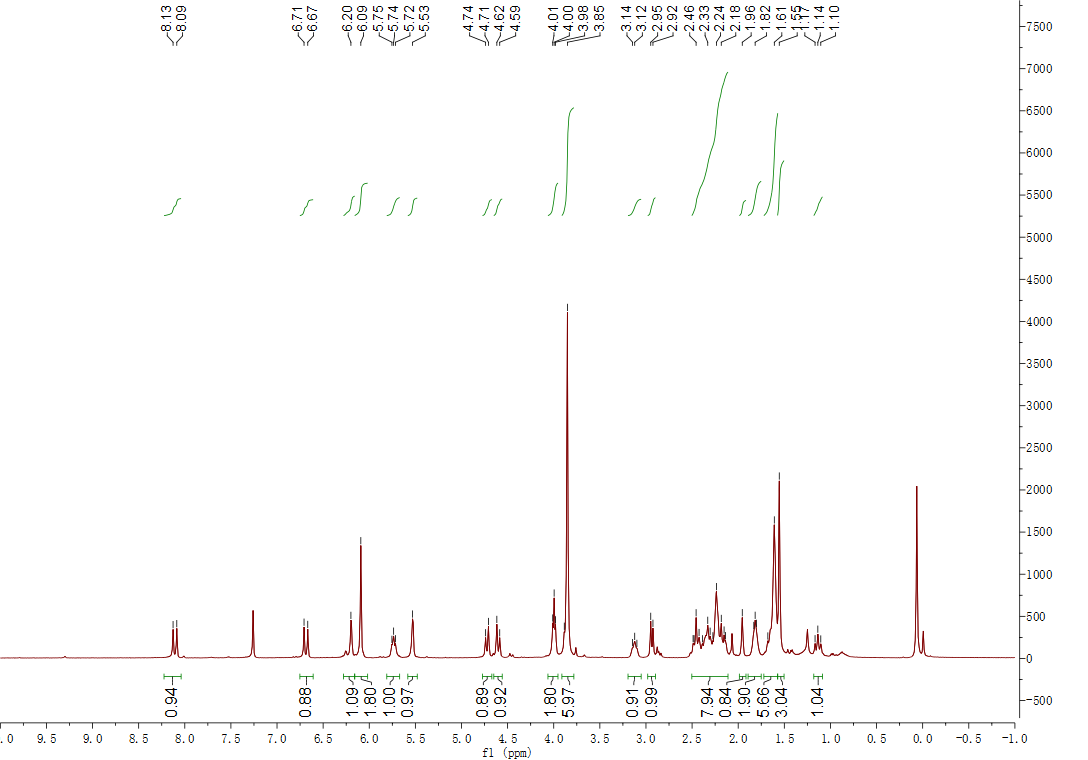


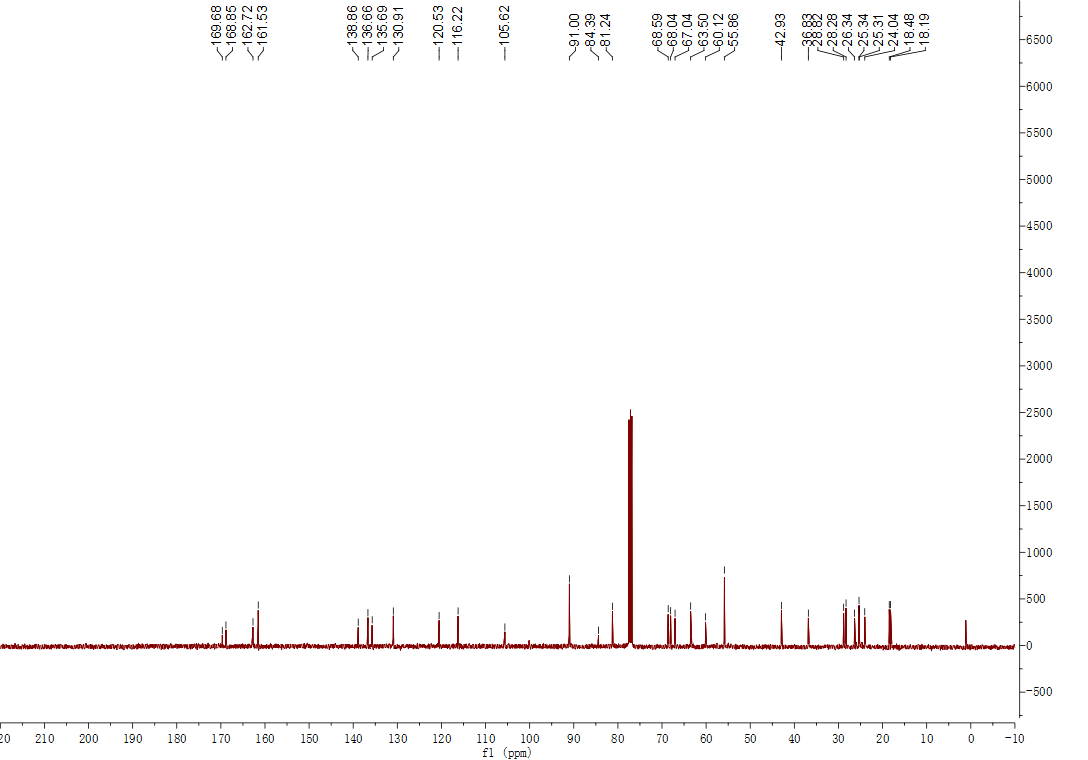

_
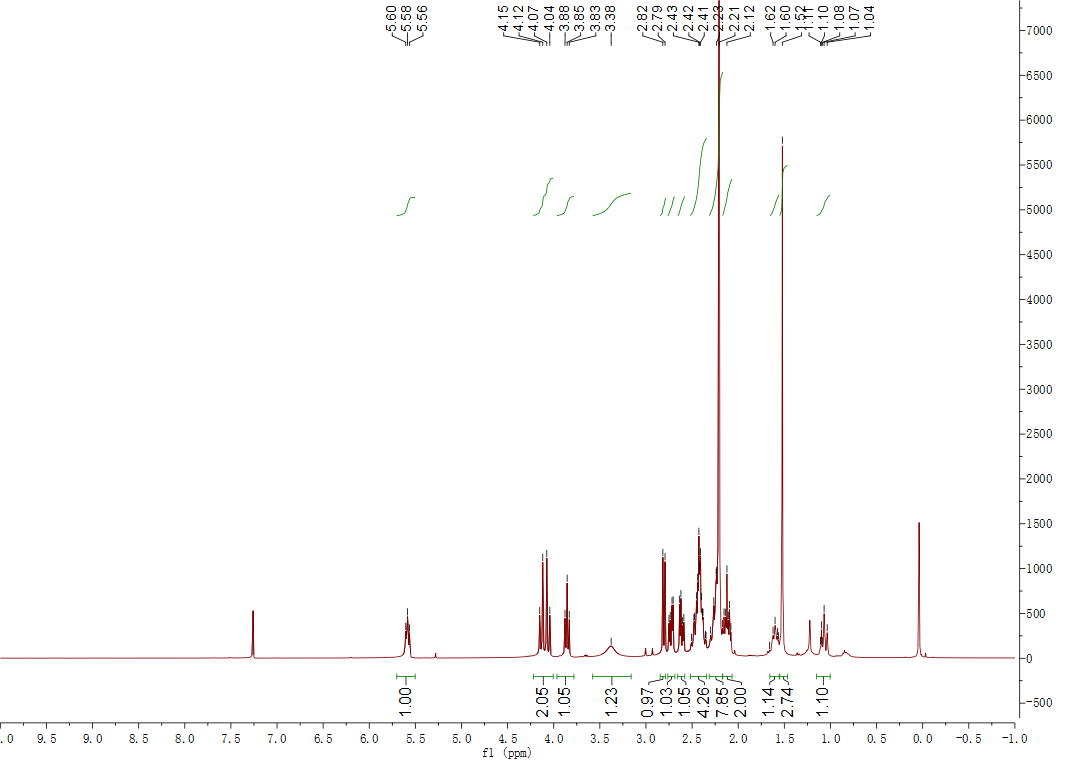
_

_
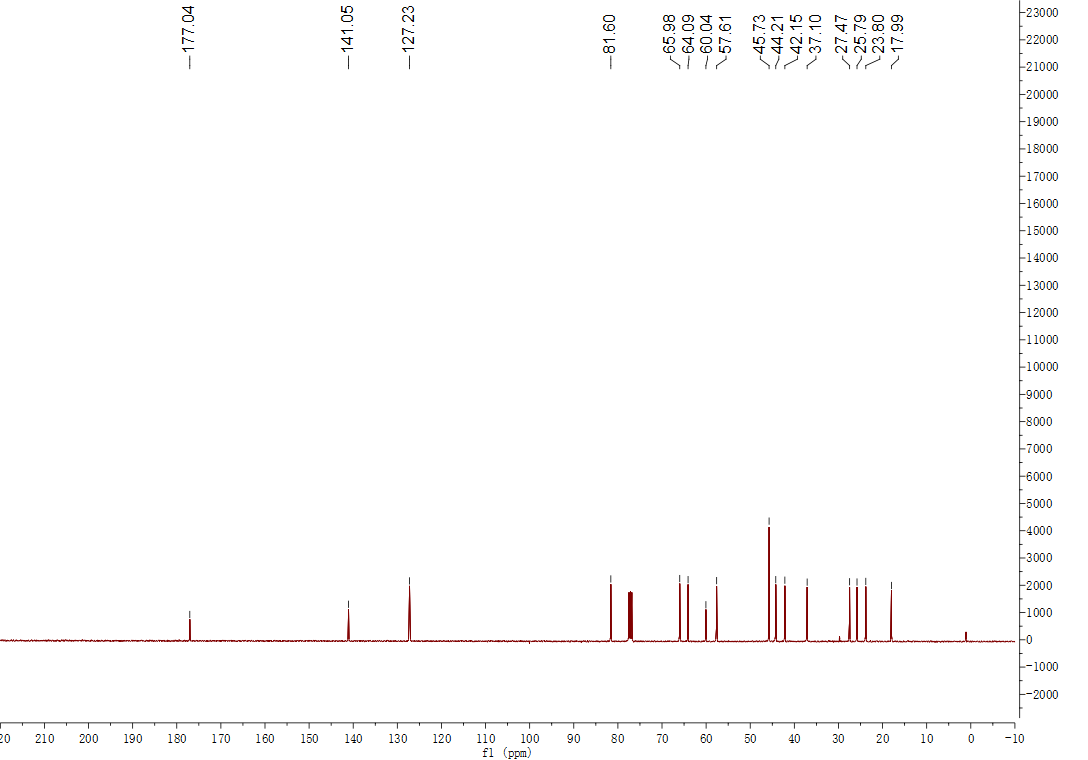
_

_
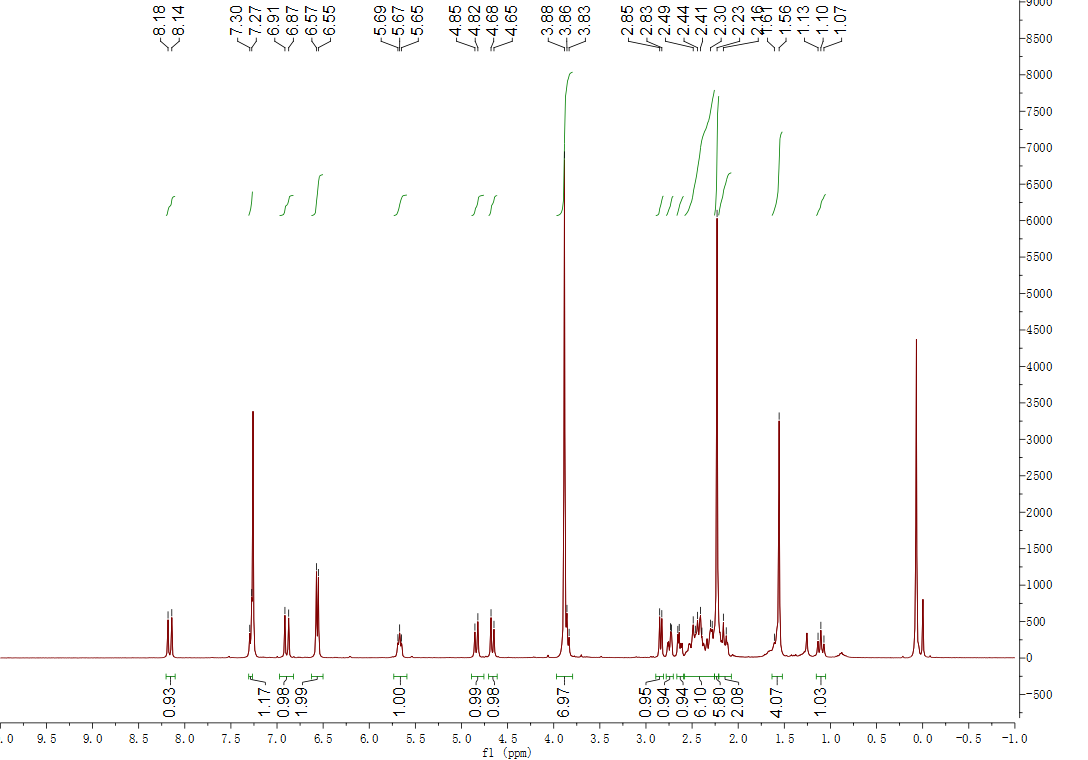
_

_
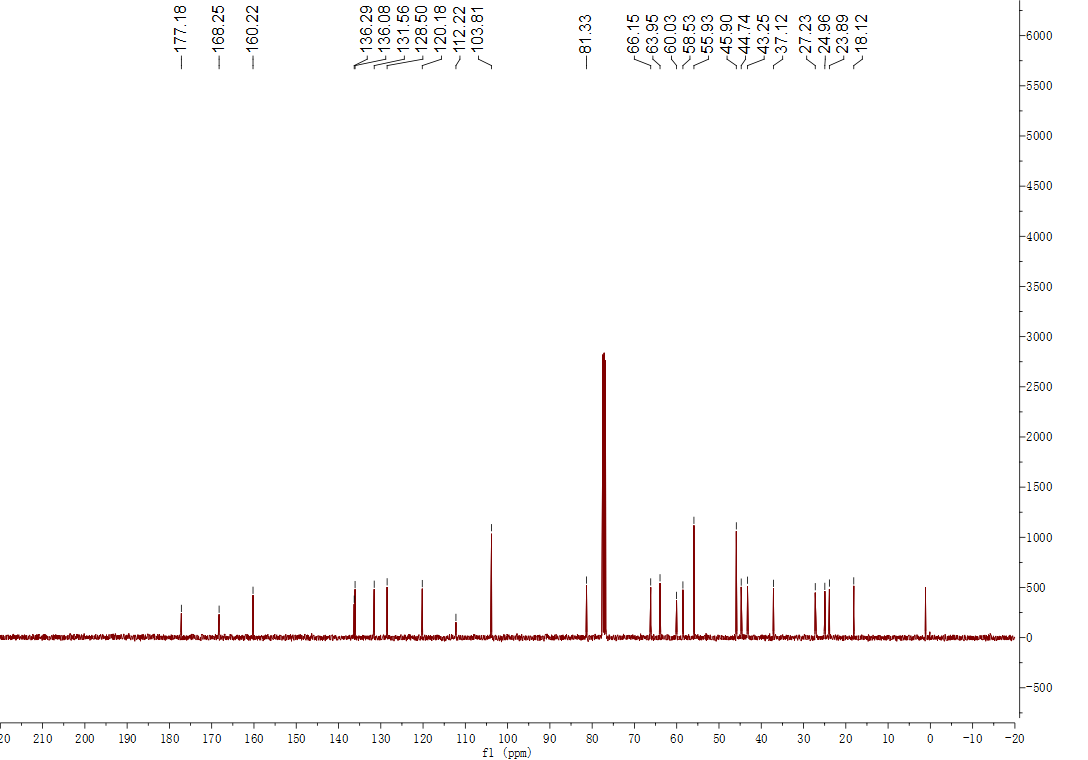
_


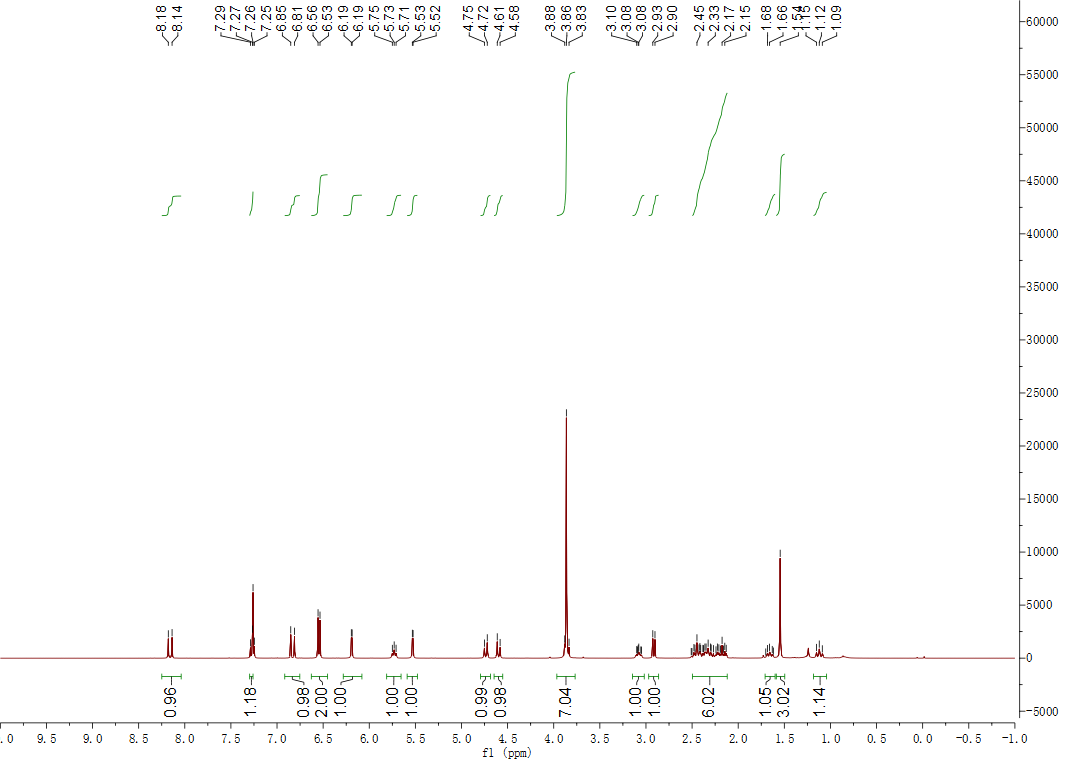


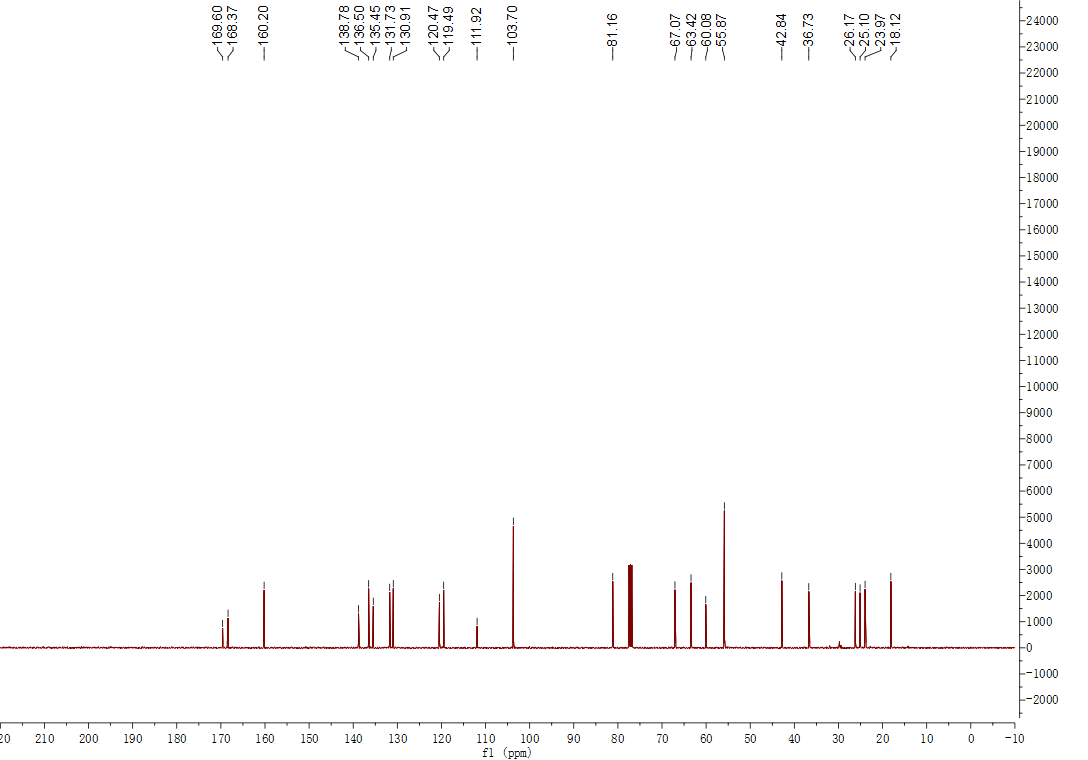

**
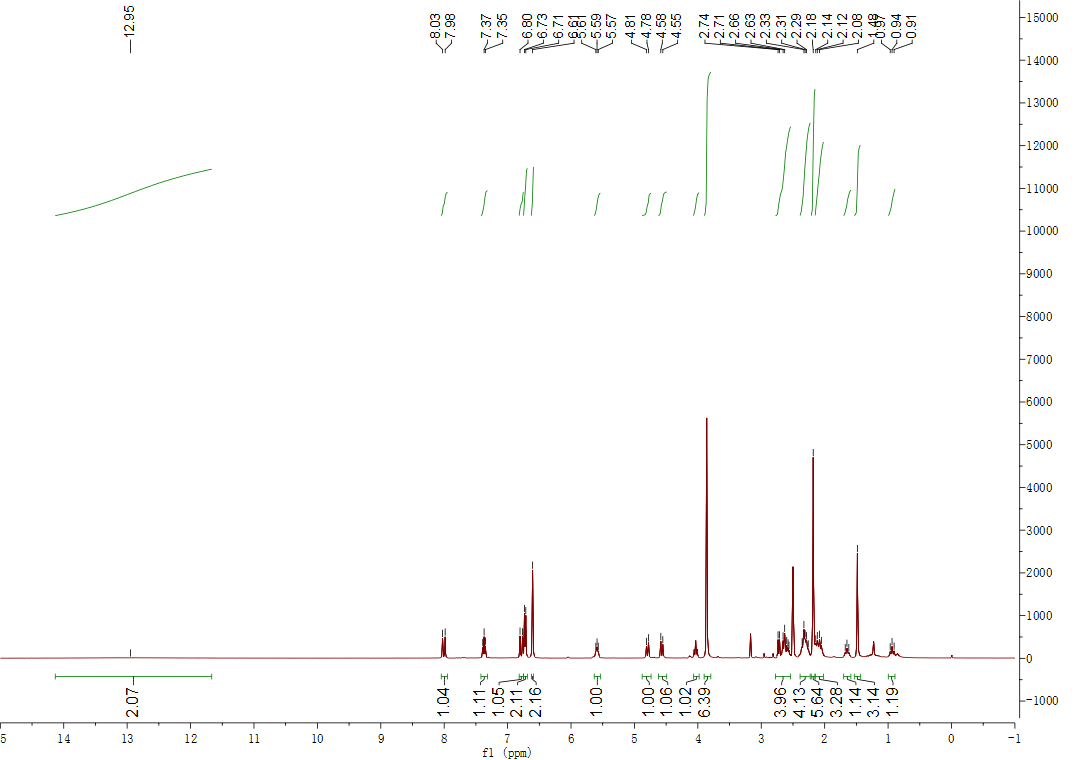
**

**
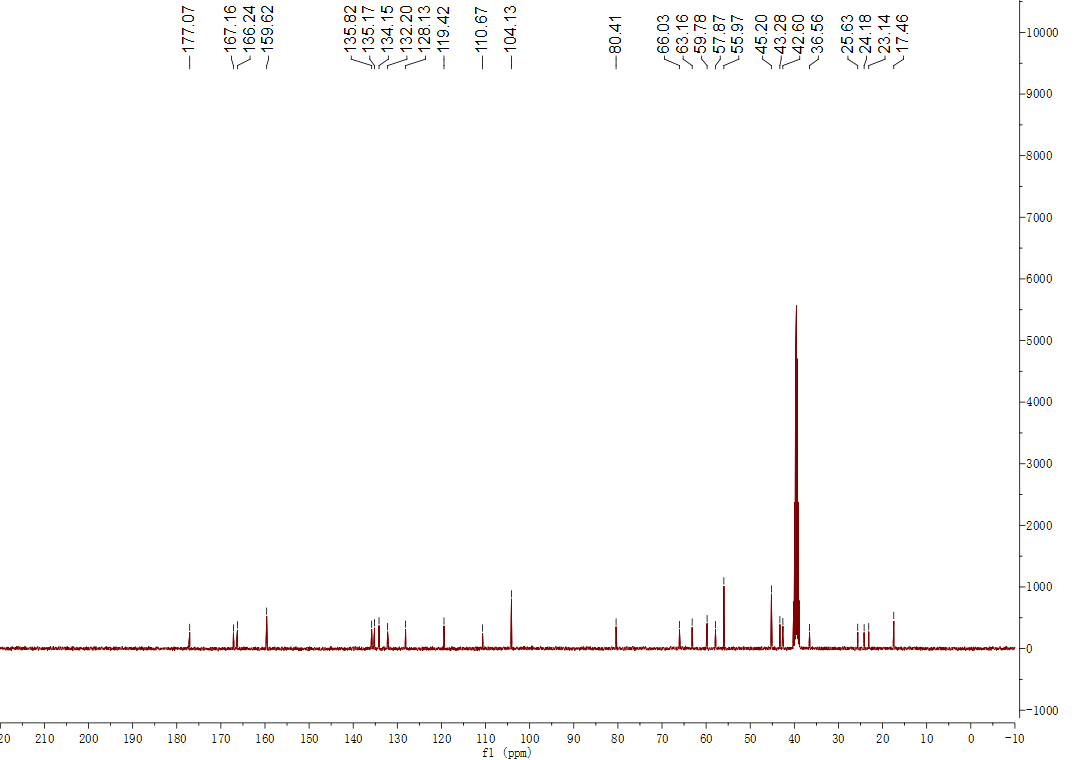
**


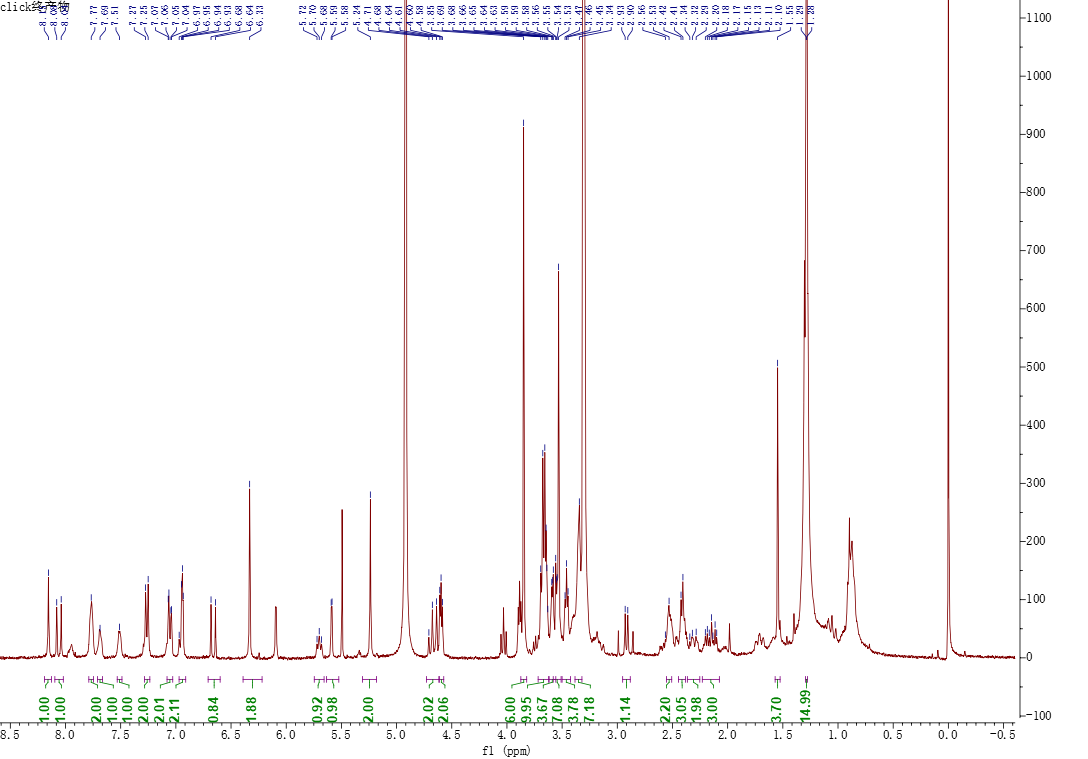

Supplement: Supplementary file 1 — Additional file 1. NMR copies of compounds. [file 13045_2020_1016_MOESM1_ESM.docx]
